# Particle–Hole Symmetry Breaking in Nitrogen-Decorated Triphenylmethyl Radical Emitters

**DOI:** 10.1021/acs.jpca.5c08128

**Published:** 2026-01-31

**Authors:** Alessio Graziano Rizzo, Marco Tommaso Barreca, Francesco Di Maiolo

**Affiliations:** Department of Chemistry, Life Science and Environmental Sustainability, Università di Parma, Parma 43124, Italy

## Abstract

Organic radical emitters have recently emerged as promising alternatives to conventional singlet emitters, as they circumvent spin-statistical limits and can, in principle, achieve unity internal quantum efficiency in OLEDs. Here, we study the photophysics of a series of nitrogen-decorated triphenylmethyl radicals using the Pariser–Parr–Pople (PPP) model within the Restricted Active Space Configuration Interaction (RASCI) framework. By exploiting the PPP particle–hole difference operator introduced in *J. Phys. Chem. C* **2024**, *128*, 18158–18169, we quantify particle–hole symmetry breaking and relate it to the oscillator strength of the first absorption band. Systematic nitrogen substitution at the meta positions of the phenyl rings leads to increasingly bright doublet states. We further show that an effective difference operator value can be computed using ground-state DFT energies, enabling a fast and practical screening protocol for identifying potentially emissive radicals. Our results provide simple design rules and predictive indicators for engineering bright organic radicals through controlled particle–hole symmetry breaking.

## Graphical abstract

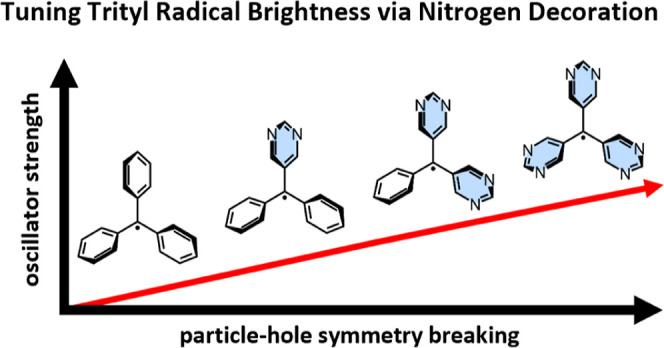

## 1 Introduction

Organic light-emitting diodes (OLEDs) have conventionally employed closed-shell organic molecules or phosphorescent complexes to achieve high electroluminescence efficiencies. In such systems, light emission typically originates from singlet or triplet exciton recombination. Because the ground state of most organic emitters is a singlet, the radiative decay of triplet excitons is spin-forbidden, resulting in significant non-emissive losses. Consequently, in first-generation fluorescent OLEDs, triplet excitons act as energy traps, limiting the internal quantum efficiency (IQE) to approximately 25% according to spin statistics. To overcome this intrinsic limit, various molecular design strategies have been developed to render triplet excitons emissive and thereby approach the theoretical IQE limit of 100%. One approach employs phosphorescent emitters containing heavy-metal centers, which facilitate efficient intersystem crossing and triplet harvesting.^1–3^ Another exploits thermally activated delayed fluorescence (TADF),^4,5^ including its hybrid implementation in hyperfluorescent architectures that couple TADF sensitizers with fluorescent dopants.^6–8^ In addition, multiresonant charge-transfer (MR-CT) emitters have emerged as efficient systems combining high color purity with narrowband emission.^4,9–14^ More recently, open-shell organic radicals have attracted increasing attention as a distinct class of emissive materials for OLEDs.^15–29^ Their characteristic unpaired electrons give rise to an alternative emission mechanism involving transitions between doublet excited and doublet ground states. This spin-allowed process circumvents the singlet–triplet energy gap and eliminates the need for intersystem crossing, thereby enabling all charge recombination events to contribute to radiative emission. Such a mechanism minimizes nonradiative losses and provides a promising route toward intrinsically efficient organic light-emitting systems.

The presence of strong electron correlation in organic radicals poses significant challenges for the accurate prediction of their excited-state properties. This complexity arises from the multiconfigurational character of many radical electronic states, which necessitates the use of ab initio methods capable of describing static and dynamic correlation effects on an equal footing. To this end, several multireference quantum-chemical approaches have been developed^18,24,30–49^ Despite their accuracy, the computational cost of these methods has so far restricted their application to relatively small molecular systems. Consequently, ground-state density functional theory (DFT) and its time-dependent extension (TDDFT) remain the methods of choice for exploring the electronic structure and excited-state behavior of larger organic radicals, in spite of their well-known theoretical limitations.^15–21,23,50–56^

In this work, we adopt an alternative approach by performing a comprehensive investigation of a series of triphenylmethyl (trityl) radicals using the Pariser–Parr–Pople (PPP) model Hamiltonian, which provides an effective framework for incorporating electron correlation in π-conjugated systems. The PPP model has demonstrated remarkable success in describing both ground-state and excited-state properties of a wide range of organic molecules.^57–67^ More recently, renewed interest in this model has emerged owing to its ability to account for systems featuring an inverted singlet–triplet (InveST) energy gap^68–71^ and to reproduce doublet emission processes in both planar and nonplanar organic radicals.^72–75^ Although originally developed for planar π-conjugated hydrocarbons, the PPP model has undergone several important extensions to enhance its range of applicability. Nonplanar geometries can be described through additional terms that account for torsional and out-of-plane distortions,^29,67,73,76–79^ while the inclusion of heteroatoms can be treated through parameter optimization guided by experimental data.^69,74,80–84^ These adaptations substantially expand the model versatility, allowing it to accurately capture the electronic properties of open-shell systems that combine nonplanarity and heteroatom substitution, two key features of contemporary organic radical emitters.^15–25^

A key feature of the PPP model, that is only approximately preserved in ab initio all-electron Hamiltonians, is its inherent particle–hole (p–h) symmetry. This symmetry finds an almost exact realization in the class of alternant hydrocarbons, which provide a simple yet powerful molecular framework for its manifestation.^58,67,72,74,85–90^ In an alternant hydrocarbon, the carbon atoms can be partitioned into two distinct sets through a “starring” procedure, in which every other carbon atom is marked with a star such that no two starred atoms are directly bonded (see trityl radical in Figure 1). This construction ensures that starred atoms are bonded exclusively to unstarred atoms, and vice versa. Within the PPP framework, this gives rise to a characteristic pattern in the electronic structure: the molecular orbitals (MOs) occur in symmetric pairs of equal magnitude and opposite sign in energy. In systems with an even number of atoms, the negative-energy orbitals are filled in the ground state, while their positive-energy counterparts remain empty, forming bonding–antibonding orbital pairs. In contrast, alternant hydrocarbons containing an odd number of atoms possess a nonbonding orbital exactly at zero energy, which is singly occupied in the open-shell ground state (see trityl radical in Figure 1). Importantly, this singly occupied molecular orbital (SOMO) is localized entirely on one of the two sublattices—specifically, the one containing the larger number of atoms—as illustrated in the Supporting Information Section S4. Strict p–h symmetry in the PPP model assumes a homonuclear carbon lattice. The inclusion of heteroatoms disrupts this symmetry by altering the on-site energies, thereby introducing an intrinsic asymmetry in the energies of the bonding and antibonding orbitals.

**Figure 1:**
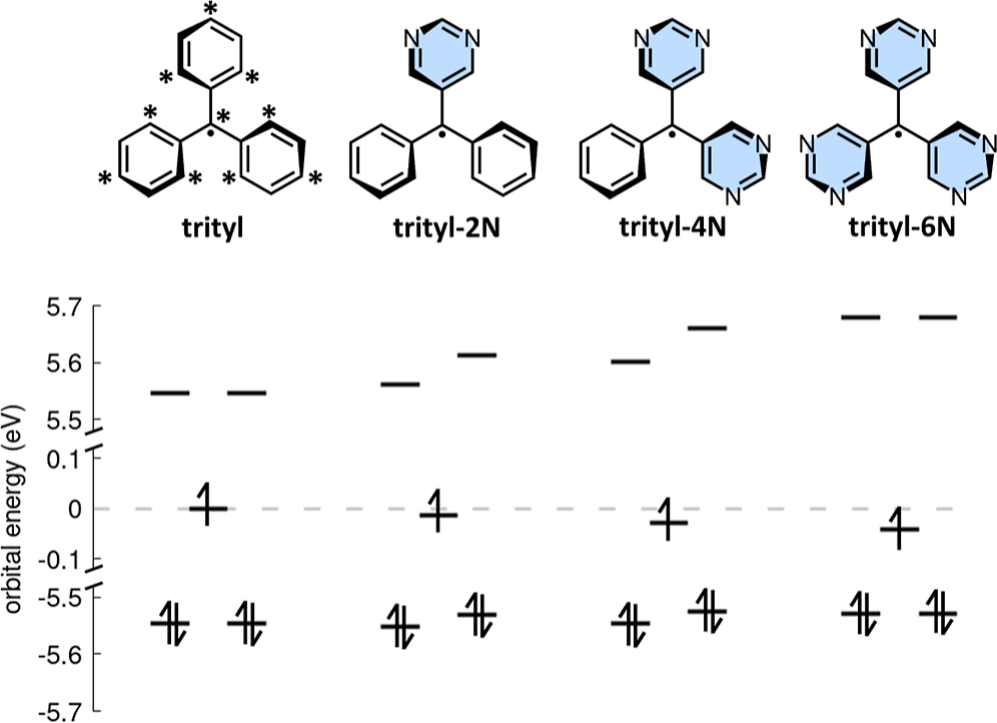
Molecular structures of four prototypical propeller-shaped organic radicals—triphenylmethyl (trityl), diaza-triphenylmethyl (trityl-2N), tetraaza-triphenylmethyl (trityl-4N), and hexaaza-triphenylmethyl (trityl-6N)—together with their corresponding frontier molecular orbital energies calculated at the PPP–Hartree–Fock level. Each arrow denotes one electron. PPP model parameters are discussed in the main text.

In this work, we use the PPP model to study p–h symmetry breaking and its effect on doublet emission in several trityl radicals differently decorated with nitrogen atoms. The PPP model is not intended to give quantitatively exact predictions for specific molecular systems.^68,91^ Instead, its strength lies in providing a conceptually transparent and computationally efficient framework capable of capturing the essential qualitative trends associated with electron correlation in π-conjugated molecules.

## 2 Methods

The PPP model represents one of the simplest yet powerful theoretical frameworks for studying electron correlation in π-conjugated systems. Similar to the Hückel model, it restricts the electronic description to the 2p_*z*_ atomic orbitals oriented perpendicular to the molecular framework at each atomic site. In contrast to the Hückel approach, however, the PPP Hamiltonian explicitly incorporates electron–electron interactions in the zero differential overlap (ZDO) approximation, which neglects interatomic overlap between 2p_*z*_ orbitals while retaining on-site and long-range Coulomb repulsion terms. The PPP model Hamiltonian, formulated in the real-space atomic orbital basis, takes the following form

1 $$\begin{array}{l} {Ĥ}_{PPP}=\sum_{\mu }\varepsilon_{\mu }{\mathrm{n̂}}_{\mu }-\sum_{\mu \nu ,\nu >\mu }\sum_{\sigma }t_{\mu \nu }\left({â}_{\mu \sigma }^{†}{â}_{\nu \sigma }+{â}_{\nu \sigma }^{†}{â}_{\mu \sigma }\right)\\ +\sum_{\mu }U_{\mu }{\mathrm{n̂}}_{\mu ↑}{\mathrm{n̂}}_{\mu ↓}+\sum_{\mu \nu ,\nu >\mu }V_{\mu \nu }\left(Z_{\mu }-{\mathrm{n̂}}_{\mu }\right)\left(Z_{\nu }-{\mathrm{n̂}}_{\nu }\right) \end{array}$$

Here, ${â}_{\mu \sigma }^{†}$ and ${â}_{\mu \sigma }$ are the creation and annihilation operators, respectively, for an electron of spin σ at atomic site μ, and ${\mathrm{n̂}}_{\mu }=\sum_{\sigma }{â}_{\mu \sigma }^{†}{â}_{\mu \sigma }$ is the corresponding electron number operator. The parameters appearing in the first line of the Hamiltonian are defined as follows: ε_μ_ denotes the on-site orbital energy, and *t*_μν_ represents the hopping integral between atomic sites μ and ν. In the PPP framework, hopping is limited to atoms connected through a σ bond. The second line of the Hamiltonian accounts for electron–electron interactions. *U*_μ_ is the on-site Coulomb repulsion between two electrons occupying the same atomic orbital, while *V*_μν_ describes the intersite Coulomb interaction between electrons located on different atoms. The parameter *Z*_μ_ represents the nuclear charge associated with site μ after removal of the π-electrons; for both carbon and aza-nitrogen atoms, *Z*_μ_ = 1. Within the Ohno approximation, *V*_μν_ is expressed as^61,64,92^

2 $$V_{\mu \nu }=\frac{e^{2}}{4\pi \varepsilon_{0}}{\left[r_{\mu \nu }^{2}+{\left(\frac{\varepsilon_{r}e^{2}}{4\pi \varepsilon_{0}\left(U_{\mu }+U_{\nu }\right)}\right)}^{2}\right]}^{-1/2}$$

where the relative dielectric constant, ε_*r*_, is set to 2, representing a typical organic environment.^64^

For computational convenience, the molecular geometries were idealized by fixing all bond angles to 120° and all bond lengths to 1.4 Å. This simplified π-skeleton is consistent with the standard PPP parametrization and enables an exclusive focus on the electronic consequences of π-conjugation. A quantitative comparison with DFT-optimized ab initio geometries is provided in Section S3 of the Supporting Information. Although the PPP model was originally formulated for planar π-conjugated hydrocarbons, it can be readily extended to nonplanar architectures by incorporating torsional effects into the electronic hopping term. In the case of the trityl radical derivatives, DFT-optimized geometries (Tables S3–S9) reveal a propeller-like conformation, with dihedral angles of approximately 32° between the central carbon and each of the three peripheral phenyl rings. Accordingly, the hopping integrals *t*_μν_ connecting the central carbon atom to the phenyl substituents are modeled to depend on the torsional angle θ through a cosine modulation

3 $$t_{\mu \nu }\to t_{\mu \nu }\left(\theta \right)=t_{\mu \nu }\;\cos\theta$$

where θ = 32°.

To enable direct comparison with conventional quantum-chemical approaches, it is convenient to rewrite the PPP Hamiltonian in the molecular orbital representation. Within the Hartree–Fock (HF) approximation, the PPP Hamiltonian in eq 1 reduces to the one-electron Fock operator

4 $${\mathrm{F̂}}_{PPP}=\sum_{\mu }\left(\varepsilon_{\mu }+J_{\mu \mu }-K_{\mu \mu }\right){\mathrm{n̂}}_{\mu }+\sum_{\mu \nu ,\mu \neq \nu }\left(-t_{\mu \nu }-K_{\mu \nu }\right)\sum_{\sigma }\left({â}_{\mu \sigma }^{†}{â}_{\nu \sigma }+{â}_{\nu \sigma }^{†}{â}_{\mu \sigma }\right)$$

By adopting the ZDO approximation, the Coulomb and exchange operators take the forms

5 $$J_{\mu \mu }=\sum_{\lambda =1}^{N}\left(P_{\lambda \lambda }-Z_{\lambda }\right)V_{\lambda \mu },,K_{\mu \nu }=\left(P_{\mu \nu }/2-Z_{\nu }\delta_{\mu \nu }\right)V_{\mu \nu }$$

where the density matrix element is defined as

6 $$P_{\mu \nu }=2\sum_{k=1}^{SOMO-1}c_{k\mu }c_{k\nu }+c_{SOMO,\mu }c_{SOMO,\nu }$$

In eq 6, the first term runs over the doubly occupied MOs in the ground-state configuration, whereas the second term corresponds to the SOMO. The coefficients *c*_*k*μ_ represent the expansion of the *k*-th MO in the atomic orbital basis (see Supporting Information, Section S4). The Fock operator is solved self-consistently through the variational minimization of the total electronic energy. Convergence of the density matrix elements typically requires about 50 HF iterations.

After obtaining the HF MOs for the ground-state configuration |*g*⟩, excited configurations are generated by promoting one, two, three, or more electrons from occupied to virtual MOs, giving rise to single, double, triple, and higher-order excitations. Our configuration interaction (CI) basis explicitly includes the ground-state configuration |*g*⟩ together with the excited configurations, and the PPP Hamiltonian is then expressed in this basis and diagonalized using the CI formalism. However, in this representation, the Hamiltonian becomes considerably less sparse than in the real-space basis. To make the problem numerically tractable while retaining the essential electron-correlation effects, we employ the Restricted Active Space Configuration Interaction (RASCI) approach, recently adapted to the PPP framework.^70^ In this methodology,^46,47^ the HF MOs are partitioned into three subspaces, namely RAS1, RAS2, and RAS3, arranged in order of increasing orbital energy. RAS1 contains predominantly occupied orbitals, RAS2 includes both occupied and low-lying virtual orbitals, and RAS3 comprises higher-energy virtual orbitals. The simplest RASCI variant, in which all possible configurations within RAS2 are considered, corresponds to a complete active space CI (CASCI). The method can be systematically extended by introducing excitations between subspaces through the hole–particle approximation, wherein a fixed number of holes are allowed in RAS1 and an equivalent number of excited particles in RAS3. For instance, the RASCI­(h,p) scheme includes single excitations either from RAS1 to RAS2 or from RAS2 to RAS3,^93^ while RASCI­(h,p,hp) additionally accounts for direct excitations from RAS1 to RAS3. The accuracy of the RASCI treatment depends critically on the orbital selection and electron distribution among the three subspaces. However, as shown below, when the RAS2 space comprises five electrons distributed over five MOs, the RASCI­(h,p,hp) results for the trityl radical and its aza-substituted derivatives reproduce those obtained at the higher-level RASCI­(h,p,hp,2h,2p) treatment, while significantly reducing the computational cost.

The molecular excited states |*f*⟩ obtained from the diagonalization of the PPP–RASCI Hamiltonian in a space that also includes the ground-state configuration |*g*⟩, are subsequently used to compute the optical spectra. The corresponding electric dipole moment operator is given by

μ⃗^=μ̂xi⃗+μ̂yj⃗=∑μ(Zμ−n̂μ)(xμi⃗+yμj⃗) 7

where *x*_μ_ and *y*_μ_ denote the Cartesian coordinates of site μ in the molecular plane. Absorption spectra are obtained as

8 $$A\left(\omega \right)\propto \omega \sum_{f}{\left|{\mathrm{\mu ⃗}}_{fg}\right|}^{2}\exp\left[\frac{{\left(\omega -\omega_{fg}\right)}^{2}}{2\sigma^{2}}\right]$$

where the sum extends over all excited eigenstates, ω_*fg*_ = *E*_*f*_ – *E*_*g*_ denotes the transition energy, and μ⃗fg=⟨f|μ⃗^|g⟩ is the corresponding transition dipole moment. Each transition is represented by a Gaussian band shape with a line width of σ = 0.1 eV. The transition energies and dipole moments determine the oscillator strength associated with the |*g*⟩ → |*f*⟩ excitation^94^

9 $$f_{fg}=\frac{2}{3}\frac{m_{e}}{\hbar e^{2}}\omega_{fg}{\left|{\mathrm{\mu ⃗}}_{fg}\right|}^{2}$$

where *m*_*e*_ is the electron mass and *e* the electron charge. All calculations reported in this work were performed with an in-house PPP-RASCI code written in Fortran.

## 3 Results & Discussion

### 3.1 Trityl, Trityl-2N, Trityl-4N, and Trityl-6N as Model Systems

In this study, we begin by focusing on the four triphenylmethyl radicals depicted in Figure 1. The parent triphenylmethyl radical (trityl) is an odd-alternant hydrocarbon showing a propeller-like geometry with *D*_3_ symmetry. Trityl holds a prominent place in the history of organic chemistry, being recognized as the first persistent radical ever discovered.^25,95–97^ Experimentally, its emission spectrum features a lowest-energy peak centered at approximately 2.38 eV.^98,99^ Upon nitrogen substitution, a variety of aza-decorated trityl derivatives have been synthesized and characterized. Most studies on aza-decorated trityl derivatives have so far focused on systems bearing nitrogen atoms in the *para* positions of the peripheral phenyl rings. Among these, the polychlorinated pyridine-diphenylmethyl motif, in which a single *para*-carbon is replaced by nitrogen, yields the *para*-substituted pyridyl radical known as PyBTM.^52^ Subsequent syntheses produced the bispyridyl-phenylmethyl (bisPyTM)^17^ and trispyridyl-methyl (trisPyM)^100^ derivatives, where nitrogen atoms occupy the *para* positions of two and three phenyl rings, respectively. In these systems, the emission maxima progressively red-shift with increasing nitrogen substitution, reflecting the enhanced electron-withdrawing character of the pyridyl units, shifting from 2.12 to 1.91 eV and further to 1.77 eV as the number of nitrogen atoms increases from one to three in CH_2_Cl_2_. More recently, in 2021, Matsuoka, Kusamoto, and co-workers reported an aza-decorated polychlorinated trityl radical incorporating a *meta*-pyridyl group (metaPyBTM).^101^ Interestingly, this species exhibits photophysical properties that closely resemble those of the corresponding trityl radical without nitrogen substitution, with an emission maximum at 2.17 eV in CH_2_Cl_2_, while displaying a remarkable solid-state luminescence at room temperature, an effect not observed for the *para*-substituted analogue.

The PPP parameters for carbon atoms are well established and broadly transferable among a wide range of π-conjugated systems.^57,61,64,102,103^ In this work, the carbon on-site energy is set to zero, with an on-site electron–electron repulsion of *U*_*C*_ = 11.26 eV and a nearest-neighbor C–C hopping integral of *t* = −2.4 eV. In contrast to carbon, there is no universally accepted set of PPP parameters for nitrogen atoms.^60–64,102,104–110^ In this work, unless otherwise specified, we adopt an on-site electron–electron repulsion of *U*_*N*_ = 15.5 eV, following ref 69, and a site energy of ε_*N*_ = – 1.5 eV, similarly to the value used in ref 64. The C–N hopping integrals are set equal to those of the C–C bonds. This parametrization yields doublet excitation energies for the aza-decorated trityl radicals that are in close agreement with ab initio CASSCF/QD-NEVPT2 results using DFT-optimized geometries (see Supporting Information Section S2).

Accurately describing the potentially strong multiconfigurational character of electronic states in organic radicals is essential for a reliable representation of their excitation spectra and photophysical behavior. The PPP model, with its minimal active space, is particularly well suited for probing the role of higher-order excited configurations in such open-shell systems. Within this framework, the PPP-RASCI approach efficiently eliminates energetically negligible configurations by restricting the number of holes and particles in the lower and higher HF-MOs, respectively. This constraint significantly reduces the overall configuration space, leading to a good balance between accuracy and computational cost. We first defined a subset of MOs to span the active RAS2 space, where all possible electron excitations are considered (i.e., equivalent to a CASCI treatment). This active space was chosen to include the most chemically relevant orbitals for the radicals under investigation. For the trityl radical, the RAS2 space comprises the doubly degenerate HOMO, the SOMO, and the doubly degenerate LUMO, thus corresponding to 5 electrons in 5 orbitals (see Figure 1). The same (5,5) active space was employed for the aza-decorated trityl derivatives. As illustrated in Figure 2, panel a, the CASCI treatment overestimates the transition energy for the parent trityl radical with respect to experiment. In particular, while the experimentally observed absorption peak lies at approximately 2.4 eV,^98^ the PPP–CASCI approach predicts a transition energy of 3.28 eV, corresponding to an overestimation of about 0.9 eV. To improve upon these results, we employed the RASCI hole–particle hierarchy, which includes additional excitations involving the remaining orbitals in the RAS1 and RAS3 subspaces. In the RASCI­(h,p) scheme, single excitations are allowed from RAS1 into RAS2 and from RAS2 into RAS3, while in RASCI­(h,p,hp) direct single excitations from RAS1 into RAS3 are also included. The latter brings the calculated transition energies into much closer agreement with the experimentally observed absorption energy of the parent trityl radical. A further refinement of dynamic correlation is achieved in the RASCI­(h,p,hp,2h,2p) extension, which includes double excitations from RAS1 into RAS2 and from RAS2 into RAS3, albeit at the expense of a substantially larger configuration space and higher computational cost. Considering the trade-off between efficiency and accuracy, the RASCI­(h,p,hp) level was therefore adopted for the exploratory studies presented in this work. Since this improved agreement is evident for trityl, where reliable experimental data are available, the same PPP-RASCI­(h,p,hp) approach was consistently applied to the aza-decorated derivatives shown in panels b–d. Corresponding transition energies obtained from the ab initio CASSCF­(5,5)/QD-NEVPT2 method are reported in Section S2 of the Supporting Information.

**Figure 2:**
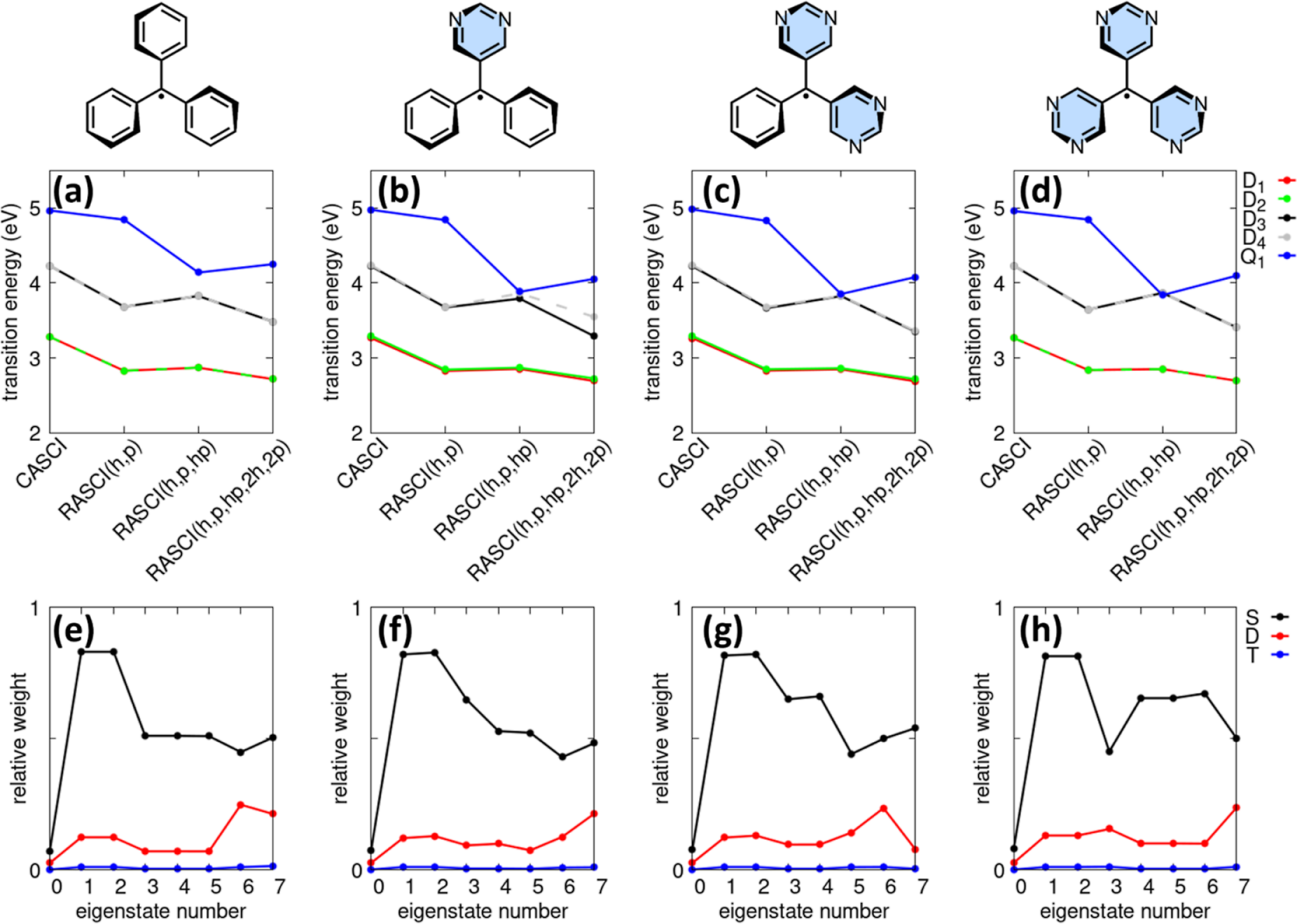
Excitation energies of the first few electronic states of trityl (panel a), trityl-2N (panel b), trityl-4N (panel c), and trityl-6N (panel d) at various theoretical levels, together with the relative contributions of singly (S), doubly (D), and triply (T) excited configurations for the first four doublet and quartet eigenstates of trityl (panel e), trityl-2N (panel f), trityl-4N (panel g), and trityl-6N (panel h) calculated at the PPP-RASCI­(h,p,hp) level. Model parameters are specified in the main text.

Within this framework, the impact of the CI space is particularly pronounced for the lowest quartet state Q_1_. As illustrated in panels a–d, CASCI and RASCI­(h,p) yield very similar transition energies for Q_1_, whereas a substantial reduction is observed upon moving to the RASCI­(h,p,hp) level. For the parent trityl radical, inclusion of direct hole-particle excitations from RAS1 into RAS3 lowers the Q_1_ transition energy by approximately 0.7 eV, while for the aza-decorated trityl radicals the corresponding reduction amounts to about 1 eV. Analysis of the CI wave function shows that at the RASCI­(h,p,hp) level the Q_1_ state acquires a significant contribution from configurations involving one hole in RAS1 and one particle in RAS3, accounting for roughly 35% of the total weight in trityl and increasing to nearly 50% in the nitrogen-substituted derivatives. These configurations are absent in both CASCI and RASCI­(h,p), explaining the pronounced stabilization of the quartet state upon inclusion of the hp excitation class. Extending the CI space further to RASCI­(h,p,hp,2h,2p) does not lead to a significant additional change in the Q_1_ transition energy, indicating that the dominant correlation effects for this state are already captured at the RASCI­(h,p,hp) level.

Panels (e–h) show the relative contributions of single (S), double (D), and triple (T) excitations to the lowest eight eigenstates of trityl, trityl-2N, trityl-4N, and trityl-6N. Excited configurations have a negligible influence on the ground state of all four molecules, which remains predominantly described (∼90%) by the Hartree–Fock determinant. In the lowest excited states, single excitations provide the dominant contribution—80–85% for D_1_ and D_2_—while double excitations account for 10–15% within the first five excited doublet states.

A comparison between the PPP-RASCI­(h,p,hp) calculations—performed with a (5,5) RAS2 active space—and the ab initio CASSCF­(5,5)/QD-NEVPT2 results was carried out for the trityl radical and its nitrogen-decorated derivatives (see Supporting Information Section S2). As expected for a semiempirical Hamiltonian, the agreement between PPP and QD-NEVPT2 is reasonable, though not exact. For the parent trityl radical, the ab initio transition energies exceed the PPP predictions by roughly 0.1 eV. For the aza-decorated derivatives, the discrepancy increases slightly, with the D_1_ and D_2_ energies lying about 0.2–0.3 eV above the corresponding PPP-RASCI values. The oscillator strengths predicted by the PPP model are systematically underestimated relative to the CASSCF/QD-NEVPT2 values – a well-known limit of the PPP Hamiltonian.^91^ Despite this quantitative shortcoming, the PPP calculations reliably capture the qualitative trend, namely the progressive increase in oscillator strength as the number of aza-nitrogen atoms on the phenyl rings is raised.

The lowest excited state of trityl transforms as *E*, and because the ground state belongs to the *A*_2_ representation, the *A*_2_ → *E* transition is symmetry-allowed within the *D*_3_ point group. Nevertheless, it remains prohibited by p–h symmetry, yielding a vanishing transition dipole moment. We remark that the faint signal detected experimentally near 2.38 eV^98,99^ can be rationalized once inductive effects are incorporated into the PPP framework. Indeed, inductive contributions relax the strict p–h symmetry of the PPP model (see Supporting Information Section S6).^111^ However, to keep the model as transparent and minimally parametrized as possible, we do not include these inductive effects in the remainder of the paper and focus exclusively on the effects arising from nitrogen substitution. In panels (b–d), introducing an increasing number of aza nitrogen atoms at the meta positions of the phenyl rings produces no substantial change in the excitation energies. Although nitrogen substitution systematically modifies the MO energies—leading to a decreasing HOMO-SOMO gap and an increasing SOMO-LUMO gap (see Figure 1)—this does not translate into a net shift of the lowest electronic excitations. The nature of the relevant vertical excitations is found to be essentially unchanged across all systems: the lowest doublet state D_1_ is predominantly described by a near-equal mixture of HOMO → SOMO and SOMO → LUMO configurations, while D_2_ mainly consists of comparable contributions from HOMO–1 → SOMO and SOMO → LUMO+1 excitations. This orbital character is conserved for trityl, trityl-2N, trityl-4N, and trityl-6N. As a result, while the individual HOMO-SOMO and SOMO-LUMO energy gaps evolve with increasing nitrogen content, the overall HOMO–LUMO and (HOMO–1)–(LUMO+1) gaps involved in the D_1_ and D_2_ excitations remain nearly constant, leading to unchanged excitation energies.

For trityl-2N and trityl-4N, the lowered symmetry (from *D*_3_ to *C*_2*v*_) lifts the degeneracy between the D_1_ and D_2_ states, whereas this degeneracy is restored in trityl-6N, which again belongs to the *D*_3_ point group. Notably, in all three aza-substituted systems, the lowest doublet states acquire finite oscillator strength, reflecting the progressive breakdown of p–h symmetry induced by nitrogen doping. Finally, across all four radicals, the first quartet state Q_1_ remains well above the lowest doublet excitations and is only minimally influenced by the aza nitrogens introduced into the phenyl framework.

The difference operator introduced in ref 90 can be used to estimate the degree of p–h symmetry breaking. It is defined as the difference between the original PPP Hamiltonian in eq 1 and its p–h–transformed counterpart (see Supporting Information Section S5 for the derivation)

10 $$\mathrm{D̂}=\sum_{\mu }\left[U_{\mu }+2\varepsilon_{\mu }\right]\left({\mathrm{n̂}}_{\mu }-1\right)$$

The term inside the square brackets remains constant in the absence of heteroatoms. The absolute value of the ground-state expectation value |⟨D̂⟩|=|⟨g|D̂|g⟩| serves as a direct indicator of the degree to which p–h symmetry is broken, specifically, it reflects the imbalance between the HOMO–SOMO and SOMO–LUMO energy gaps, which in turn modulates the optical transition probabilities. In the following, we examine how |⟨D̂⟩| varies with the aza-nitrogen on-site energy for trityl-2N, trityl-4N, and trityl-6N. The resulting trends in |⟨D̂⟩| are then compared with the integrated oscillator strength of the lowest absorption band, arising from the nearly degenerate D_1_ and D_2_ states (separated by ∼0.02 eV), and with the energies of the frontier PPP–HF molecular orbitals.

A series of spectra computed at the PPP-RASCI­(h,p,hp) level is shown in Figure 3, panels (a), (e), and (i). For each radical, the calculations were carried out by scanning the aza-nitrogen on-site energy ε_*N*_ from −4.5 eV up to 0 eV, while maintaining *U*_*N*_ = 15.5 eV fixed. By covering this interval—from strongly electronegative nitrogen to the hypothetical limit in which nitrogen has the same site energy as carbon—the role played by the nitrogen atoms in shaping the electronic properties of the decorated trityl radicals becomes evident.

**Figure 3:**
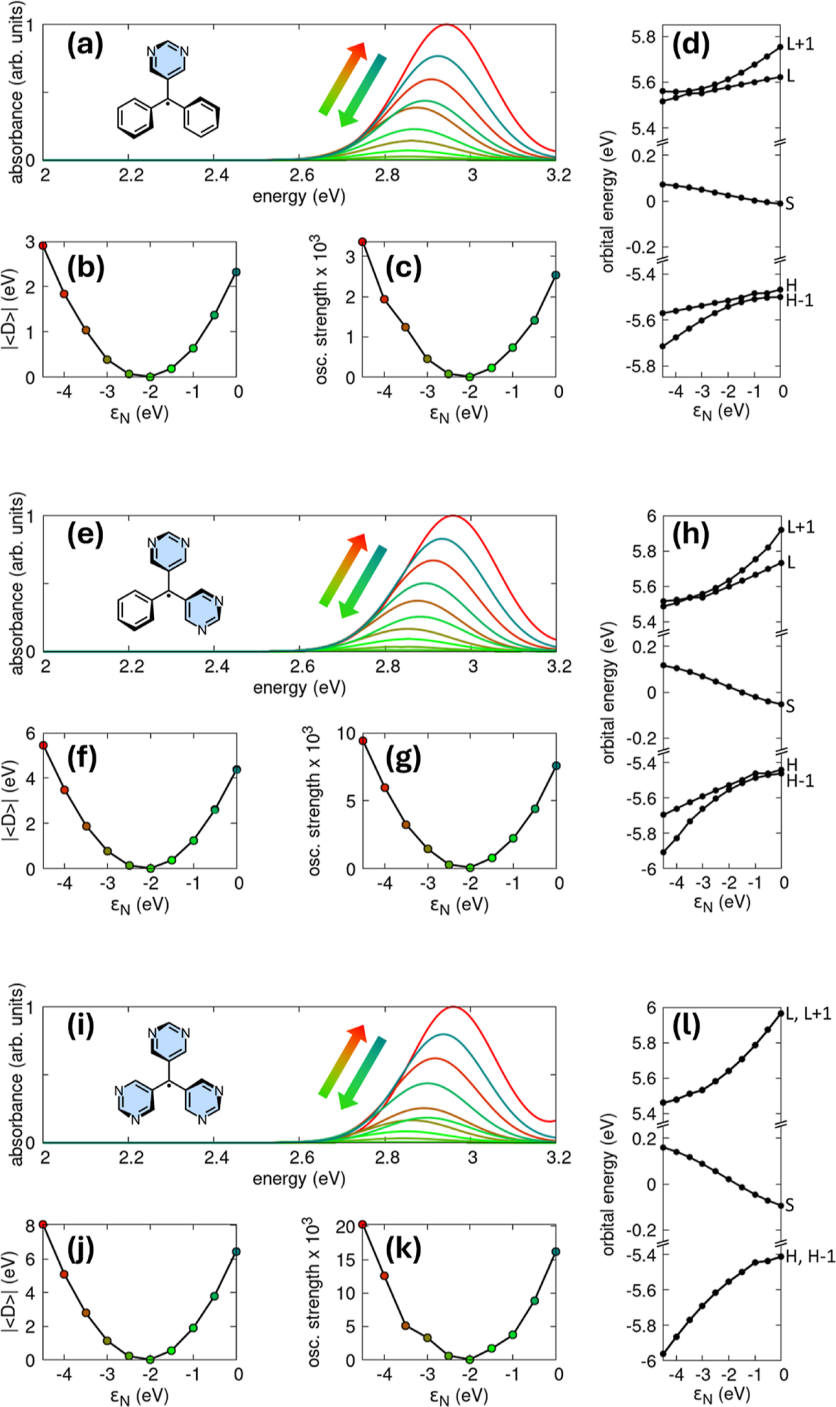
Particle–hole symmetry and optical response of trityl-2N (a–d), trityl-4N (e–h), and trityl-6N (i–l). (a) Absorption spectra of trityl-2N: colored traces correspond to different ε_*N*_ values (color scheme consistent with panels b and c). All spectra are normalized to the maximum absorbance of the most intense curve; (b) Expectation value of the difference operator evaluated for the trityl-2N ground state as a function of ε_*N*_; (c) Oscillator strength associated with the lowest bright doublet state of trityl-2N for various ε_*N*_ values; (d) Frontier PPP–HF MOs energies of trityl-2N plotted vs ε_*N*_ (H = HOMO, S = SOMO, L = LUMO); (e-h) Same as in panels (a–d), respectively, for trityl-4N; (i–l) Same as in panels (a–d) for trityl-6N. All calculations were performed at the PPP-RASCI­(h,p,hp) level. Other parameters are provided in the main text.

For trityl-2N (panel (a)), increasing the electron-withdrawing character of the nitrogen atoms (i.e., making ε_*N*_ more negative) produces a modest spectral evolution: the absorption maximum undergoes a slight red shift as ε_*N*_ is varied from 0 to −2 eV, followed by a mild blue shift in the range −4.5 eV < ε_*N*_ < −2.5 eV. Panel (b) shows that the absolute value of the ground-state expectation value of the p–h difference operator initially decreases, reaching a minimum at ε_*N*_ = −2 eV, and then increases again as the nitrogen orbital is further stabilized. This behavior can be rationalized by examining eq 10: at ε_*N*_ = −2 eV, the term inside the square brackets evaluates to 11.5 eV for nitrogen and 11.26 eV for carbon, making the p–h asymmetry minimal and suppressing the oscillator strength (panel c). Deviating from ε_*N*_ = −2 eV enhances p–h symmetry breaking; consequently, |⟨D̂⟩| increases and the oscillator strength of the lowest doublet transition rises sharply. The trends in panel (d), displaying the frontier PPP–HF molecular orbital energies as a function of ε_*N*_, further support this interpretation. At ε_*N*_ = −2 eV, the HOMO–SOMO and SOMO–LUMO gaps are almost identical, signaling nearly perfect p–h symmetry, whereas moving away from this value progressively lifts this balance. Notably, the nitrogen site energy exerts opposite effects on the MOs adjacent to the SOMO: HOMO–1, HOMO, LUMO, and LUMO+1 are stabilized as ε_*N*_ becomes more negative, while the SOMO is destabilized. As a result, the HOMO–SOMO and SOMO–LUMO energy gaps differ.

For trityl-4N and trityl-6N (panels (e) and (i)), the absorption spectra display the same qualitative evolution observed for trityl-2N as ε_*N*_ is varied. The main absorption band undergoes a slight red shift when ε_*N*_ moves from 0 to −2 eV, followed by a gradual blue shift as ε_*N*_ approaches −4.5 eV. The behavior of the difference operator (panels (f) and (j)) and of the oscillator strength associated with the lowest absorption band (panels (g) and (k)) mirrors the trends already described for trityl-2N: |⟨D̂⟩| reaches a minimum near ε_*N*_ = −2 eV and increases as p–h symmetry is progressively broken for more positive or more negative values of ε_*N*_. What changes, however, is the magnitude of these quantities. For a given ε_*N*_, both |⟨D̂⟩| and the oscillator strength are larger in trityl-4N and even more pronounced in trityl-6N, reflecting the stronger overall perturbation introduced by multiple aza nitrogen atoms. The frontier PPP–HF orbital energies shown in panels (h) and (l) also reveal a steeper and stronger modulation as a function of ε_*N*_, although the qualitative pattern remains consistent with that identified for trityl-2N—namely, stabilization of HOMO–1, HOMO, LUMO, and LUMO+1 with increasingly negative ε_*N*_, accompanied by a destabilization of the SOMO. In trityl-6N, the recovery of *D*_3_ point-group symmetry results in pairs of degenerate orbitals, specifically HOMO–1/HOMO and LUMO/LUMO+1.

The trends in |⟨D̂⟩| and in the oscillator strength observed for trityl-2N, trityl-4N, and trityl-6N as the aza-nitrogen PPP site energy is varied clearly highlight the prominent role of heteroatoms in disrupting p–h symmetry. Figure 4 maps the dependence of |⟨D̂⟩| and of the oscillator strength on the parameters ε_*N*_ and *U*_*N*_. The green marker identifies the trityl reference point, where the system consists solely of carbon atoms. Moving away from this point by introducing electron-withdrawing atoms at the meta positions of the phenyl rings—i.e., by decreasing ε_*N*_ and increasing *U*_*N*_—leads to a progressive enhancement of p–h symmetry breaking, reflected in the growing magnitude of the average difference operator. Consistently, the color map on the right reveals an accompanying increase in the oscillator strength of the *A*_2_ → *E* transition. The red marker denotes the parameter set corresponding to trityl-6N. For completeness, analogous maps for systems where only one or two phenyl rings are substituted with electron-withdrawing atoms are provided in Supporting Information Section S7, exhibiting the same overall trends.

**Figure 4:**
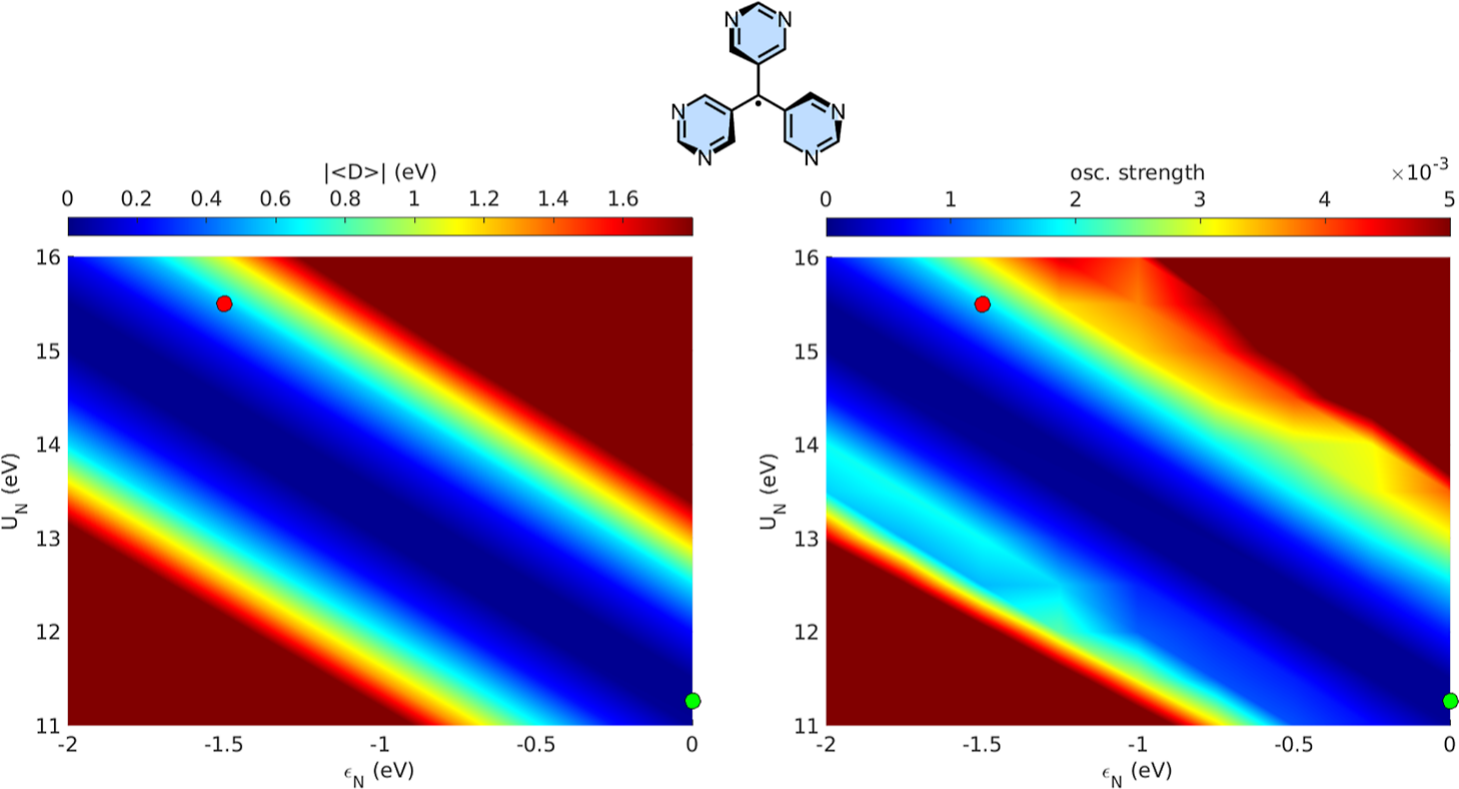
Color map on the left depicts how |⟨D̂⟩| changes across the (*U*_*N*_, ε_*N*_) parameter space, while the map on the right illustrates the corresponding variation in the oscillator strength of the *A*_2_ → *E* transition. The green marker identifies the parameter set characteristic of trityl, whereas the red marker denotes that of trityl-6N. All calculations were carried out at the PPP-RASCI­(h,p,hp) level using the model parameters specified in the main text.

### 3.2 Extending the Analysis to Other Aza-Substituted Trityl Radicals

We progressively substituted the meta positions of the phenyl rings in the trityl radical with an increasing number of aza-nitrogen atoms, as illustrated in Figure 5. Despite this growing degree of heteroatom incorporation, the main absorption band remains essentially pinned at ∼2.86 eV (panel a). A notable trend emerges when examining p–h symmetry breaking. Even the introduction of a single meta-position nitrogen in trityl-1N is sufficient to produce a measurable effect, yielding |⟨D̂⟩|≃0.08 eV (panel c). Adding a second nitrogen atom in trityl-2N increases this value to roughly 0.19 eV. Inspection of panel c suggests that each aza nitrogen contributes approximately 0.11 eV to |⟨D̂⟩| , in an almost additive fashion. The lowest-energy absorption band arises from transitions to the D_1_ and D_2_ states, which lie only ∼0.02 eV apart in all systems. The oscillator strength integrated over these two transitions (panel b) increases as more nitrogen atoms are introduced, although not in a uniform manner. A useful comparison can be made between the 1N/3N and 2N/4N pairs. In the fully asymmetric 1N and 3N radicals (*C*_1_ symmetry), the two lowest doublets involve excitations that feel the presence of the nitrogen atom(s); in other words, both D_1_ and D_2_ experience p–h symmetry breaking. In contrast, trityl-2N and trityl-4N retain *C*_2*v*_ symmetry, which preserves a distinction between frontier MOs localized on nitrogen-substituted and unsubstituted rings (see PPP-HF MOs in Supporting Information Section S4). In these systems, D_1_ is mainly composed of excitations centered on the carbon-only ring(s), which feel a weaker p–h symmetry breaking perturbation, whereas D_2_ is dominated by excitations localized on the nitrogen-bearing rings. This creates a balance: in the *C*_1_ species fewer nitrogen atoms are present, but both transitions are influenced by them, while in the *C*_2*v*_ species more nitrogen atoms are present overall, yet only one transition (D_2_) feels their full effect. This compensation naturally explains the comparable oscillator strengths observed for trityl-1N and trityl-2N, as well as for trityl-3N and trityl-4N. We note that an alternative and synthetically motivated distribution of nitrogen atoms—placing one aza nitrogen on each phenyl ring—leads to the same qualitative trends in excitation energies, oscillator strengths, and p–h symmetry breaking. These results are discussed in detail in Section S8 of the Supporting Information.

**Figure 5:**
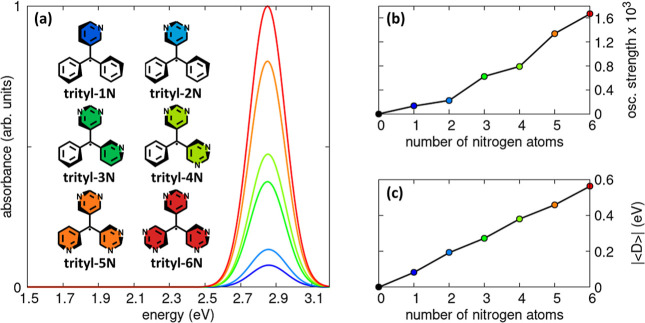
Effect of progressively introducing aza-nitrogen atoms at the meta positions of the trityl radical. Panel (a) shows the computed absorption spectra, panel (b) reports the oscillator strength integrated over the 0–3.2 eV energy window, and panel (c) displays the absolute value of the ground-state expectation of the difference operator for six trityl derivatives bearing an increasing number of meta-substituted aza nitrogens on the three phenyl rings. In panel (a), all spectra are normalized to the maximum absorbance of the most intense curve. In panels (b) and (c), the black marker denotes the values corresponding to unsubstituted trityl radical. Model parameters are provided in the main text. All calculations were carried out at the PPP-RASCI­(h,p,hp) level.

The analysis above underscores that both the PPP model and |⟨D̂⟩| serve as powerful tools for finding radicals in which p–h symmetry is strongly disrupted, an essential characteristic for achieving highly efficient doublet emitters. We recall that p–h symmetry is only approximate in all-electron Hamiltonians. In contrast, the half-filled PPP model imposes p–h symmetry by design. Whether this symmetry survives in an all-electron description depends on how closely the actual electronic structure mirrors the assumptions built into the PPP framework. As molecular systems deviate from these idealized PPP conditions, the degree of p–h symmetry that remains in all-electron treatments diminishes accordingly.

As discussed in ref 90, the difference operator defined in eq 10 can be linked to total molecular energies obtained from quantum-chemical methods. This connection becomes clear when inspecting the structure of the D̂ operator: it depends on the on-site electron density, weighted by the local electron–electron repulsion *U*_μ_, together with twice the on-site energy ε_μ_, and these contributions are summed over the whole molecule. This viewpoint suggests that ground-state DFT total energies for the radical in its different charge states can be used to define an effective molecular electron–electron repulsion energy, *U*_eff_ = *E*(trityl^–^) + *E*(trityl^+^) – 2*E*(trityl), which corresponds to the difference between the ionization potential and the electron affinity.^113,114^ Similarly, an effective on-site energy can be extracted from the ionization potential of the radical as ε_eff_ = *E*(trityl) – *E*(trityl^+^). Using these two quantities, an effective difference operator can be defined as |*D*_eff_| = *U*_eff_ + 2ε_eff_. The same reasoning applies to the trityl radicals substituted with aza-nitrogen atoms. In Figure 6, we report both |⟨D̂⟩| values computed at the PPP-RASCI­(h,p,hp) level and those obtained from DFT-level total molecular energies. Although the numerical agreement is not exact, the qualitative trend is accurately captured: |⟨D̂⟩| increases consistently with the number of aza nitrogens introduced on the phenyl rings.

**Figure 6:**
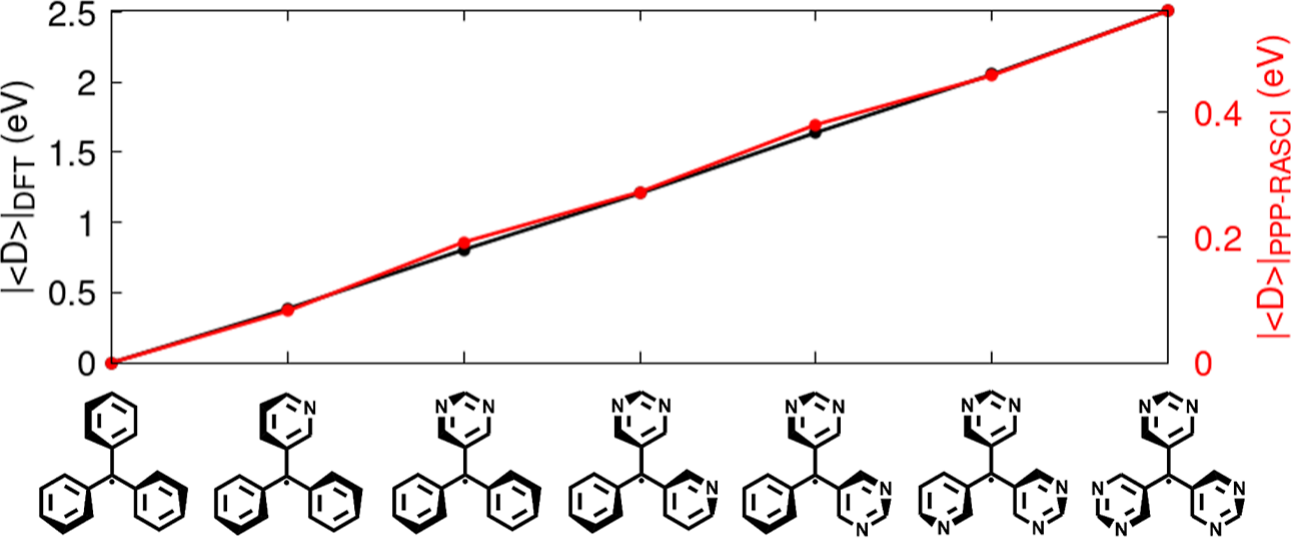
Comparison of |⟨D̂⟩| at DFT (black curve) and PPP-RASCI (red curve) levels. Values of |⟨D̂⟩| for the same series of aza-decorated trityl radicals discussed in Figure 5 are reported here as obtained from PPP–RASCI­(h,p,hp) and from DFT total-energy calculations. The PPP–RASCI values correspond to those previously shown in Figure 5, panel (c), and are based on the model parameters specified in the main text. For the DFT calculations, we employed the UBHandHLYP functional in combination with the 6-31G­(d,p) basis set, as implemented in Gaussian16,^112^ using the DFT-optimized geometries of the neutral radicals forms. To allow a direct comparison between the two approaches, the DFT-derived |⟨D̂⟩| values were rescaled with respect to the trityl reference, for which |⟨D̂⟩|=6.35 eV.

## 4 Conclusions

Radical emitters have the potential to achieve 100% internal quantum efficiency in OLED applications, as their emission arises entirely from spin-allowed doublet-to-doublet transitions. This ensures that all generated excitons can contribute to light emission, unlike singlet emitters, which are constrained by exciton spin statistics. The emission color of doublet emitters can be precisely tuned through molecular design, necessitating computational studies to identify the most efficient species. However, from a theoretical perspective, strong electron correlation in these systems complicates the calculation of their excited state properties. Although multiconfigurational approaches are typically employed, their high computational cost makes them unsuitable for rapid screening and exploratory studies. Consequently, the PPP model is a logical choice, offering a simple framework for describing interacting electrons in π-conjugated systems. Moreover, since p–h symmetry is an exact symmetry of the PPP Hamiltonian, this model is ideal for studying its effect on the arrangement of radical MOs and the influence of chemical substitution.

In Figure 2, the PPP analysis showed that doubly excited configurations account for 10–15% within the first five excited doublet states of four representative trityl radicals—trityl, trityl-2N, trityl-4N, and trityl-6N. For the parent trityl radical, the lowest optical transition is permitted by point-group symmetry but remains forbidden due to p–h symmetry, which renders this species unsuitable for OLED applications. Once nitrogen atoms are introduced, however, p–h symmetry is progressively disrupted, and the first excited doublet becomes optically allowed, as observed in trityl-2N, trityl-4N, and trityl-6N.

To quantify p–h symmetry breaking more rigorously, we made use of the difference operator D̂ (cf. eq 10). The exploratory analysis presented in Figures 3 and 4 examines how variations in the nitrogen PPP on-site energy ε_*N*_ and in the electron–electron repulsion *U*_*N*_ affect both the degree of p–h symmetry breaking and the brightness of the lowest doublet state in trityl-2N, trityl-4N, and trityl-6N. Pronounced symmetry breaking emerges when heteroatoms are introduced whose values of *U*_μ_ + 2ε_μ_ differ substantially from those characteristic of carbon atoms.

In Figure 5, we begin from the parent trityl radical and progressively introduce nitrogen atoms at the meta positions of the phenyl rings. Even the incorporation of a single heteroatom is enough to break p–h symmetry, yielding |⟨D̂⟩|≃0.08 eV. As additional nitrogens are introduced, this symmetry breaking strengthens: each new nitrogen contributes roughly 0.11 eV to the overall value of |⟨D̂⟩| . A comparable trend appears in the oscillator strength, though not in a uniform manner. The largest increases occur when the first nitrogen atom is introduced on a given phenyl ring, since this breaks the molecular symmetry most strongly and affects both D_1_ and D_2_, which lie only ∼0.02 eV apart in all studied radicals. Consequently, sharp rises in oscillator strength appear for trityl-1N, trityl-3N, and trityl-5N. By contrast, adding a second nitrogen to the same ring produces only a modest additional enhancement: although more nitrogen atoms are present overall, only one of the two low-lying doublets fully feels the additional perturbation.

Because double excitations are computationally demanding, their routine use in all-electron calculations becomes impractical for broad exploratory studies. Our analysis, however, shows that the absolute value of the ground-state average difference operator already serves as a robust indicator of p–h symmetry breaking and of the emergence of finite oscillator strength. Even though all-electron Hamiltonians do not strictly obey p–h symmetry, an effective quantity |*D*|_eff_ can still be constructed from ground-state DFT total energies. In Figure 6, we compare the ground-state values of |⟨D̂⟩| obtained at the PPP-RASCI and DFT levels for a representative set of trityl radicals. The two approaches display a strong qualitative correspondence, confirming that ground-state DFT provides a reliable framework for identifying new potentially emissive organic radicals, as anticipated in ref 90.

## Supplementary Material



## Data Availability

The data that support the findings of this study are available from the corresponding author upon reasonable request.

## Notes

Sec. S1 contains ab initio computational details; Sec. S2 collects CASSCF molecular orbitals and QD-NEVPT2 energies; Sec. S3 compares PPP-idealized and DFT-optimized geometries; Sec. S4 contains the PPP Hartree–Fock molecular orbitals; Sec. S5 presents the derivation of the difference operator; Sec. S6 collects details on the inductive effects in the trityl radical; Sec. S7 presents color maps obtained decorating the meta positions in one and two phenyl rings with electron-acceptor atoms; Sec. S8 collects results for additional nitrogen-decorated trityl radicals; Sec. S9 contains the Cartesian coordinates for the DFT-optimized geometries (PDF)

## References

[1] Baldo M. A., O’Brien D. F., You Y., Shoustikov A., Sibley S., Thompson M. E., Forrest S. R. (1998). Highly efficient
phosphorescent emission from organic electroluminescent devices. Nature.

[2] Adachi C., Baldo M. A., Thompson M. E., Forrest S. R. (2001). Nearly 100% internal
phosphorescence efficiency in an organic light-emitting device. J. Appl. Phys..

[3] Minaev B., Baryshnikov G., Agren H. (2014). Principles of phosphorescent organic
light emitting devices. Phys. Chem. Chem. Phys..

[4] Dos
Santos J. M., Hall D., Basumatary B., Bryden M., Chen D., Choudhary P., Comerford T., Crovini E., Danos A., De J., Diesing S., Fatahi M., Griffin M., Gupta A. K., Hafeez H., Hämmerling L., Hanover E., Haug J., Heil T., Karthik D., Kumar S., Lee O., Li H., Lucas F., Mackenzie C. F. R., Mariko A., Matulaitis T., Millward F., Olivier Y., Qi Q., Samuel I. D. W., Sharma N., Si C., Spierling L., Sudhakar P., Sun D., Tankelevičiutė E., Duarte Tonet M., Wang J., Wang T., Wu S., Xu Y., Zhang L., Zysman-Colman E. (2024). The Golden Age of Thermally Activated
Delayed Fluorescence Materials: Design and Exploitation. Chem. Rev..

[5] Di
Maiolo F., Phan Huu D. K. A., Giavazzi D., Landi A., Racchi O., Painelli A. (2024). Shedding light on thermally-activated
delayed fluorescence. Chemical Science.

[6] Stavrou K., Franca L. G., Danos A., Monkman A. P. (2024). Key requirements
for ultraefficient sensitization in hyperfluorescence organic light-emitting
diodes. Nat. Photonics.

[7] Deori U., Nanda G. P., Murawski C., Rajamalli P. (2024). A perspective
on next-generation hyperfluorescent organic light-emitting diodes. Chem. Sci..

[8] Aarti A., Veettil B. P., Rodger A., Venkatesan K. (2025). Recent Progress
and Challenges in Molecular Design for Hyperfluorescent Based Organic
Light Emitting Diodes (OLEDs). Advanced Optical
Materials.

[9] Wu X., Ni S., Wang C.-H., Zhu W., Chou P.-T. (2025). Comprehensive
Review
on the Structural Diversity and Versatility of Multi-Resonance Fluorescence
Emitters: Advance, Challenges, and Prospects toward OLEDs. Chem. Rev..

[10] Shizu K., Kaji H. (2022). Comprehensive understanding
of multiple resonance thermally activated
delayed fluorescence through quantum chemistry calculations. Communications Chemistry.

[11] Kusakabe Y., Shizu K., Tanaka H., Tanaka K., Kaji H. (2024). An inverted
singlet-triplet excited state in a pentaazaphenalene derivative (5AP-N­(C12)­2). Applied Physics Express.

[12] Okumura R., Tanaka H., Shizu K., Fukushima S., Yasuda Y., Kaji H. (2024). Development of an Organic
Emitter
Exhibiting Reverse Intersystem Crossing Faster than Intersystem Crossing. Angew. Chem., Int. Ed..

[13] Pérez-Jiménez Á. J., Olivier Y., Sancho-García J. C. (2025). The Role of Theoretical
Calculations for INVEST Systems: Complementarity Between Theory and
Experiments and Rationalization of the Results. Advanced Optical Materials.

[14] Anwer M., Yin S. (2025). Recent progress and
prospects of inverted singlet-triplet energy
gap (INVEST) materials in OLEDs. Org. Electron..

[15] Peng Q., Obolda A., Zhang M., Li F. (2015). Organic Light-Emitting
Diodes Using a Neutral π Radical as Emitter: The Emission from
a Doublet. Angew. Chem., Int. Ed..

[16] Ai X., Evans E. W., Dong S., Gillett A. J., Guo H., Chen Y., Hele T. J. H., Friend R. H., Li F. (2018). Efficient
radical-based light-emitting diodes with doublet emission. Nature.

[17] Kimura S., Tanushi A., Kusamoto T., Kochi S., Sato T., Nishihara H. (2018). A luminescent
organic radical with two pyridyl groups:
high photostability and dual stimuli-responsive properties, with theoretical
analyses of photophysical processes. Chemical
Science.

[18] Guo H., Peng Q., Chen X.-K., Gu Q., Dong S., Evans E. W., Gillett A. J., Ai X., Zhang M., Credgington D., Coropceanu V., Friend R. H., Brédas J.-L., Li F. (2019). High stability and
luminescence efficiency in donor-acceptor neutral
radicals not following the Aufbau principle. Nat. Mater..

[19] He C., Li Z., Lei Y., Zou W., Suo B. (2019). Unraveling the Emission
Mechanism of Radical-Based Organic Light-Emitting Diodes. J. Phys. Chem. Lett..

[20] Abdurahman A., Hele T. J. H., Gu Q., Zhang J., Peng Q., Zhang M., Friend R. H., Li F., Evans E. W. (2020). Understanding
the luminescent nature of organic radicals for efficient doublet emitters
and pure-red light-emitting diodes. Nat. Mater..

[21] Cho E., Coropceanu V., Brédas J.-L. (2020). Organic Neutral Radical Emitters:
Impact of Chemical Substitution and Electronic-State Hybridization
on the Luminescence Properties. J. Am. Chem.
Soc..

[22] Chen Z., Li Y., Huang F. (2021). Persistent
and Stable Organic Radicals: Design, Synthesis,
and Applications. Chem..

[23] Hudson J. M., Hele T. J. H., Evans E. W. (2021). Efficient light-emitting
diodes from
organic radicals with doublet emission. J. Appl.
Phys..

[24] Gorgon S., Lv K., Grüne J., Drummond B. H., Myers W. K., Londi G., Ricci G., Valverde D., Tonnelé C., Murto P., Romanov A. S., Casanova D., Dyakonov V., Sperlich A., Beljonne D., Olivier Y., Li F., Friend R. H., Evans E. W. (2023). Reversible spin-optical interface
in luminescent organic radicals. Nature.

[25] Mizuno A., Matsuoka R., Mibu T., Kusamoto T. (2024). Luminescent Radicals. Chem. Rev..

[26] Guan J., Zhu Z., Gou Q., Wang J., Kuang Z., Zhang L., Zhang X., Ai X., Abdurahman A., Peng Q. (2025). Achieving Intrinsic Luminescence
of Pure Organic Mono- and Di-Radicals
in Aggregated States. Aggregate.

[27] Xie Y., Wu S., Zhu Z., Wang J., Kuang Z., Zhang L., Abdurahman A., Peng Q., Ai X. (2025). Stable Luminescent
Radicals with Efficient Through-Space Charge-Transfer Emission. Angew. Chem., Int. Ed..

[28] Wang X., Wang S., Ding Z., Shen L., Zhu Z., Abdurahman A., Lu G., Peng Q. (2025). Spin-Correlated Luminescence
Enabled by Bright-Dark Radical Pairing in a Diradical System. Angew. Chem., Int. Ed..

[29] Savi L., Barreca M. T., Bedogni M., Di Maiolo F. (2025). Organic Diradicals
Bridged by Inverted Singlet-Triplet Units for Optical-Spin Interfaces. J. Chem. Theory Comput..

[30] Vergés J. A., SanFabián E., Chiappe G., Louis E. (2010). Fit of Pariser-Parr-Pople
and Hubbard model Hamiltonians to charge and spin states of polycyclic
aromatic hydrocarbons. Phys. Rev. B.

[31] Marie A., Burton H. G. A. (2023). Excited States, Symmetry Breaking,
and Unphysical Solutions
in State-Specific CASSCF Theory. J. Phys. Chem.
A.

[32] Zhang S., Zhou Z., Qu Z. (2024). Diradical-Based
Strategy in Designing
Narrowband Thermally Activated Delayed Fluorescence Molecules with
Tunable Emission Wavelengths. J. Phys. Chem.
Lett..

[33] Li B., Li L., Wang Y., Peng J. (2020). CAS Calculation of the Excited States
of the Methylthio Neutral Radical and Its Ions. ACS Omega.

[34] Mesto D., Orza M., Bardi B., Punzi A., Ratera I., Veciana J., Farinola G., Painelli A., Terenziani F., Blasi D., Negri F. (2025). Luminescent
Trityl-based Diradicaloids:
A Theoretical and Experimental Assessment of Charge-Resonance in Low-Lying
Excited States. Chem.–Eur. J..

[35] Chattopadhyay S., Chaudhuri R. K., Mahapatra U. S., Ghosh A., Ray S. S. (2016). State-specific
multireference perturbation theory: development and present status. WIREs Computational Molecular Science.

[36] Smith C. E., King R. A., Crawford T. D. (2005). Coupled cluster methods including
triple excitations for excited states of radicals. J. Chem. Phys..

[37] Sengupta A., Ramabhadran R. O., Raghavachari K. (2016). Breaking a bottleneck: Accurate extrapolation
to “gold standard” CCSD­(T) energies for large open shell
organic radicals at reduced computational cost. J. Comput. Chem..

[38] Das S., Herng T. S., Zafra J. L., Burrezo P. M., Kitano M., Ishida M., Gopalakrishna T. Y., Hu P., Osuka A., Casado J., Ding J., Casanova D., Wu J. (2016). Fully Fused
Quinoidal/Aromatic Carbazole Macrocycles with Poly-radical Characters. J. Am. Chem. Soc..

[39] Huang R., Phan H., Herng T. S., Hu P., Zeng W., Dong S.-q., Das S., Shen Y., Ding J., Casanova D., Wu J. (2016). Higher Order -Conjugated Polycyclic
Hydrocarbons with Open-Shell Singlet Ground State: Nonazethrene versus
Nonacene. J. Am. Chem. Soc..

[40] Desroches M., Mayorga Burrezo P., Boismenu-Lavoie J., Peñ a Álvarez M., Gómez-García C. J., Matxain J. M., Casanova D., Morin J., Casado J. (2017). Breaking Bonds and Forming Nanographene
Diradicals with Pressure. Angew. Chem., Int.
Ed..

[41] Lu X., Lee S., Hong Y., Phan H., Gopalakrishna T. Y., Herng T. S., Tanaka T., Sandoval-Salinas M. E., Zeng W., Ding J., Casanova D., Osuka A., Kim D., Wu J. (2017). Fluorenyl Based Macrocyclic
Polyradicaloids. J. Am. Chem. Soc..

[42] Liu C., Sandoval-Salinas M. E., Hong Y., Gopalakrishna T. Y., Phan H., Aratani N., Herng T. S., Ding J., Yamada H., Kim D., Casanova D., Wu J. (2018). Macrocyclic
Polyradicaloids with Unusual Super-ring Structure and Global Aromaticity. Chem..

[43] Pérez-Guardiola A., Sandoval-Salinas M. E., Casanova D., San-Fabián E., Pérez-Jiménez A. J., Sancho-García J. C. (2018). The role
of topology in organic molecules: origin and comparison of the radical
character in linear and cyclic oligoacenes and related oligomers. Phys. Chem. Chem. Phys..

[44] Pérez-Guardiola A., Ortiz-Cano R., Sandoval-Salinas M. E., Fernández-Rossier J., Casanova D., Pérez-Jiménez A. J., Sancho-García J. C. (2019). From cyclic nanorings to single-walled
carbon nanotubes: disclosing the evolution of their electronic structure
with the help of theoretical methods. Phys.
Chem. Chem. Phys..

[45] Ni Y., Sandoval-Salinas M. E., Tanaka T., Phan H., Herng T. S., Gopalakrishna T. Y., Ding J., Osuka A., Casanova D., Wu J. (2019). [n]­Cyclo-para-biphenylmethine
Polyradicaloids: [n]­Annulene Analogs
and Unusual Valence Tautomerization. Chem..

[46] Sandoval-Salinas M. E., Carreras A., Casanova D. (2019). Triangular
graphene nanofragments:
open-shell character and doping. Phys. Chem.
Chem. Phys..

[47] Casanova D. (2022). Restricted
active space configuration interaction methods for strong correlation:
Recent developments. WIREs Computational Molecular
Science.

[48] Preethalayam P., Roldao J. C., Castet F., Casanova D., Radenković S., Ottosson H. (2024). 3,4-Dimethylenecyclobutene:
A Building Block for Design
of Macrocycles with Excited State Aromatic Low-Lying High-Spin States. Chem.–Eur. J..

[49] Ukai T., Nakata K., Yamanaka S., Kubo T., Morita Y., Takada T., Yamaguchi K. (2007). CASCI-DFT study of the phenalenyl
radical system. Polyhedron.

[50] Morita Y., Kawai J., Fukui K., Nakazawa S., Sato K., Shiomi D., Takui T., Nakasuji K. (2003). Topological
Symmetry
Control in Spin Density Distribution: Spin Chemistry of Phenalenyl-Based
Neutral Monoradical Systems. Org. Lett..

[51] Morita Y., Suzuki S., Sato K., Takui T. (2011). Synthetic organic spin
chemistry for structurally well-defined open-shell graphene fragments. Nat. Chem..

[52] Hattori Y., Kusamoto T., Nishihara H. (2014). Luminescence,
Stability, and Proton
Response of an Open-Shell (3,5-Dichloro-4-pyridyl)­bis­(2,4,6-trichlorophenyl)­methyl
Radical. Angew. Chem., Int. Ed..

[53] Diez-Cabanes V., Seber G., Franco C., Bejarano F., Crivillers N., Mas-Torrent M., Veciana J., Rovira C., Cornil J. (2018). Design of
Perchlorotriphenylmethyl (PTM) Radical-Based Compounds for Optoelectronic
Applications: The Role of Orbital Delocalization. ChemPhysChem.

[54] Tonnelé C., Casanova D. (2022). Rationalization and
tuning of doublet emission in organic
radicals. Journal of Materials Chemistry C.

[55] Shen T., Dijkstra D., Farrando-Pérez A., Boj P. G., Villalvilla J. M., Quintana J. A., Zou Y., Hou X., Wei H., Li Z., Sun Z., Díaz-García M. A., Wu J. (2023). Fused Triangulene Dimers: Facile Synthesis by Intramolecular Radical-Radical
Coupling and Application for Near-Infrared Lasers. Angew. Chem., Int. Ed..

[56] Ju C.-W., Shen Y., French E. J., Yi J., Bi H., Tian A., Lin Z. (2024). Accurate Electronic and Optical Properties
of Organic Doublet Radicals Using Machine Learned Range-Separated
Functionals. J. Phys. Chem. A.

[57] Pariser R., Parr R. G. (1953). A Semi-Empirical Theory of the Electronic
Spectra and
Electronic Structure of Complex Unsaturated Molecules. II. J. Chem. Phys..

[58] Pariser R. (1956). Theory of
the Electronic Spectra and Structure of the Polyacenes and of Alternant
Hydrocarbons. J. Chem. Phys..

[59] Pariser R. (1956). Electronic
Spectrum and Structure of Azulene. J. Chem.
Phys..

[60] Favini G., Vandoni I., Simonetta M. (1965). Calculation
of electronic spectra
of aza-benzenes and aza-naphthalenes by the Pariser-Parr-Pople method. Theoretica Chimica Acta.

[61] Albert I. D. L., Ramasesha S., Das P. K. (1991). Properties of some low-lying electronic
states in polymethineimines and poly­(2,3-diazabutadienes). Phys. Rev. B.

[62] Mukhopadhyay S., Topham B. J., Soos Z. G., Ramasesha S. (2008). Neutral and
Charged Excited States in Polar Organic Films: Origin of Unusual Electroluminescence
in Tri-*p*-tolylamine-Based Hole Conductors. J. Phys. Chem. A.

[63] Kumar M., Pati Y. A., Ramasesha S. (2012). A density
matrix renormalization
group method study of optical properties of porphines and metalloporphines. J. Chem. Phys..

[64] Thomas S., Pati Y., Ramasesha S. (2013). Linear and
nonlinear optical properties
of expanded porphyrins: A DMRG study. J. Phys.
Chem. A.

[65] Aryanpour K., Shukla A., Mazumdar S. (2014). Electron correlations
and two-photon
states in polycyclic aromatic hydrocarbon molecules: A peculiar role
of geometry. J. Chem. Phys..

[66] Bhattacharyya P., Rai D. K., Shukla A. (2020). Pariser–Parr–Pople
Model Based Configuration-Interaction Study of Linear Optical Absorption
in Lower-Symmetry Polycyclic Aromatic Hydrocarbon Molecules. J. Phys. Chem. C.

[67] Poh Y. R., Morozov D., Kazmierczak N. P., Hadt R. G., Groenhof G., Yuen-Zhou J. (2024). Alternant
Hydrocarbon Diradicals as Optically Addressable
Molecular Qubits. J. Am. Chem. Soc..

[68] Jorner K., Pollice R., Lavigne C., Aspuru-Guzik A. (2024). Ultrafast
Computational Screening of Molecules with Inverted Singlet-Triplet
Energy Gaps Using the Pariser-Parr-Pople Semiempirical Quantum Chemistry
Method. J. Phys. Chem. A.

[69] Bedogni M., Giavazzi D., Di Maiolo F., Painelli A. (2024). Shining Light on Inverted
Singlet-Triplet Emitters. J. Chem. Theory Comput..

[70] Bedogni M., Di Maiolo F. (2024). Singlet-Triplet Inversion in Triangular
Boron Carbon
Nitrides. J. Chem. Theory Comput..

[71] Fabian, M. D. ; Glaser, N. ; Solomon, G. C. The PPP model—a minimum viable parametrisation of conjugated chemistry for modern computing applications. Digital Discovery 2025. Accepted Manuscript.10.1039/D5DD00445D.

[72] Hele, T. J. H. On the electronic structure of alternant conjugated organic radicals for light-emitting diode applications. In Proceedings Volume 11799, Physical Chemistry of Semiconductor Materials and Interfaces XX; 117991A; SPIE Nanoscience + Engineering, 2021.

[73] Green J. D., Hele T. J. H. (2024). ExROPPP: Fast,
accurate, and spin-pure calculation
of the electronically excited states of organic hydrocarbon radicals. J. Chem. Phys..

[74] Shen J., Walker L. E., Ma K., Green J. D., Bronstein H., Butler K. T., Hele T. J. H. (2025). Learning
radical excited states from
sparse data. Chemical Science.

[75] Zeng J., He C.-C., Qiu S.-B., Yao Y. (2025). Orbital inversion mechanisms
hidden in strongly correlated radical systems. Appl. Phys. Lett..

[76] Ellis R. L., Kuehnlenz G., Jaffé H. H. (1972). The use of the CNDO method in spectroscopy:
VI. further n-π* transitions. Theoretica
Chimica Acta.

[77] Surján P. R., Kuzmany H. (1986). Interruption of conjugations
of polyacetylene chains. Phys. Rev. B.

[78] Hele T. J. H., Fuemmeler E. G., Sanders S. N., Kumarasamy E., Sfeir M. Y., Campos L. M., Ananth N. (2019). Anticipating Acene-Based
Chromophore Spectra with Molecular Orbital Arguments. J. Phys. Chem. A.

[79] Green J. D., Fuemmeler E. G., Hele T. J. H. (2022). Inverse molecular design from first
principles: Tailoring organic chromophore spectra for optoelectronic
applications. J. Chem. Phys..

[80] Radovic L.
R., Karra M., Skokova K., Thrower P. A. (1998). The role of substitutional
boron in carbon oxidation. Carbon.

[81] Perkins P. G., Wall D. H. (1966). Self-consistent
molecular-orbital calculations on borazines. Journal of the Chemical Society A: Inorganic, Physical, Theoretical.

[82] Jensen H., Skancke P. N., Westman S., Norin T. (1968). Semi-empirical Parameters
in pi-Electron Systems. V. The Carbonyl Group. Acta Chem. Scand..

[83] Chandra
Jha P., Krishnan A., Das P. K., Ramasesha S. (2002). Nonlinear
optical properties of linear chain phosphazenes, (PN)­x. J. Chem. Phys..

[84] Das M. (2010). Low-lying
excitations of poly-fused thiophene within Pariser-Parr-Pople model:
A density matrix renormalization group study. J. Chem. Phys..

[85] Bondeson S., Soos Z. (1979). Charge transfer transitions
in extended correlated electronic systems. Chem.
Phys..

[86] Ducasse I. R., Miller T. E., Soos Z. G. (1982). Correlated
states in finite polyenes:
Exact PPP results. J. Chem. Phys..

[87] Soos Z. G. (1983). Electronic
Structure of Ion-Radical Organic Solids and Polyenes. Isr. J. Chem..

[88] Kopp S. M., Nakamura S., Phelan B. T., Poh Y. R., Tyndall S. B., Brown P. J., Huang Y., Yuen-Zhou J., Krzyaniak M. D., Wasielewski M. R. (2024). Luminescent
Organic Triplet Diradicals
as Optically Addressable Molecular Qubits. J.
Am. Chem. Soc..

[89] Poh Y. R., Yuen-Zhou J. (2025). Enhancing
the Optically Detected Magnetic Resonance
Signal of Organic Molecular Qubits. ACS Central
Science.

[90] Dubbini M., Bonvini F., Savi L., Di Maiolo F. (2024). Turning on
Organic Radical Emitters. J. Phys. Chem. C.

[91] Misurkin I. A., Ovchinnikov A. A. (1974). The Electronic Structure of Conjugated
Systems in Terms
of the Pariser-Parr-Pole Approximation. Russ.
Chem. Rev..

[92] Ohno K. (1964). Some remarks
on the Pariser-Parr-Pople method. Theoretica
chimica acta.

[93] Casanova D., Head-Gordon M. (2009). Restricted
active space spin-flip configuration interaction
approach: theory, implementation and examples. Phys. Chem. Chem. Phys..

[94] Lakowicz, J. R. Principles of fluorescence spectroscopy, 3rd ed.; Springer: New York, 2006.

[95] Gomberg M. (1900). AN INSTANCE
OF TRIVALENT CARBON: TRIPHENYLMETHYL. J. Am.
Chem. Soc..

[96] Lewis G. N., Lipkin D., Magel T. T. (1944). The Light
Absorption and Fluorescence
of Triarylmethyl Free Radicals. J. Am. Chem.
Soc..

[97] Lankamp H., Nauta W., MacLean C. (1968). A new interpretation
of the monomer-dimer
equilibrium of triphenylmethyl- and alkylsubstituted-diphenyl methyl-radicals
in solution. Tetrahedron Lett..

[98] Bromberg A., Meisel D. (1985). Photophysics of arylmethyl radicals
at 77 K. Structure-photoreactivity
correlation. J. Phys. Chem..

[99] Schmidt J. A., Hilinski E. F. (1988). Evolution of electronically excited triphenylmethyl
radical. Picosecond preparation-pump-probe spectroscopic experiments. J. Am. Chem. Soc..

[100] Kimura S., Uejima M., Ota W., Sato T., Kusaka S., Matsuda R., Nishihara H., Kusamoto T. (2021). An Open-shell, Luminescent,
Two-Dimensional Coordination
Polymer with a Honeycomb Lattice and Triangular Organic Radical. J. Am. Chem. Soc..

[101] Matsuoka R., Kimura S., Kusamoto T. (2021). Solid-State Room-Temperature
Near-Infrared Photoluminescence of a Stable Organic Radical. ChemPhotoChem..

[102] Soos Z. G., Ramasesha S. (1984). Valence-bond
theory of linear Hubbard
and Pariser-Parr-Pople models. Phys. Rev. B.

[103] Prodhan S., Soos Z. G., Ramasesha S. (2014). Model for
triplet state engineering in organic light emitting diodes. J. Chem. Phys..

[104] Nishimoto K., Forster L. S. (1966). SCF-MO calculations
of heteroatomic
systems with the variable-β approximation. Theoretica Chimica Acta.

[105] Michl J., Koutecky J., Becker R. S., Earhart C. E. (1970). A note
on the parameters for heteroatoms in Pariser-Parr-Pople (PPP) calculations. Theoretica Chimica Acta.

[106] Hinze J., Beveridge D. L. (1971). Parametrization
of semiempirical
π-electron molecular orbital calculations. π Systems containing
carbon, nitrogen, oxygen, and fluorine. J. Am.
Chem. Soc..

[107] Zahradník R., Tesařová I., Pancíř J. (1971). Experimental
and theoretical (HMO and LCI-SCF) study of singlet-triplet transitions
in conjugated hydrocarbons and their derivatives. Collect. Czech. Chem. Commun..

[108] Griffiths J. (1982). Practical
aspects of colour prediction of organic dye
molecules. Dyes Pigm..

[109] Grossjean M. F., Tavan P. (1988). Wavelength regulation in bacteriorhodopsin
and halorhodopsin: A Pariser–Parr–Pople multireference
double excitation configuration interaction study of retinal dyes. J. Chem. Phys..

[110] Albert I.
D. L., Das P. K., Ramasesha S. (1993). Optical nonlinearities
in symmetric cyanine dyes and related systems. Journal of the Optical Society of America B.

[111] Ramasesha S., Soos Z. (1984). Magnetic and optical properties of
exact PPP states of naphthalene. Chem. Phys..

[112] Frisch, M. J. ; Trucks, G. W. ; Schlegel, H. B. ; Scuseria, G. E. ; Robb, M. A. ; Cheeseman, J. R. ; Scalmani, G. ; Barone, V. ; Petersson, G. A. ; Nakatsuji, H. ; Li, X. ; Caricato, M. ; Marenich, A. V. ; Bloino, J. ; Janesko, B. G. ; Gomperts, R. ; Mennucci, B. ; Hratchian, H. P. ; Ortiz, J. V. ; Izmaylov, A. F. ; Sonnenberg, J. L. ; Williams-Young, D. ; Ding, F. ; Lipparini, F. ; Egidi, F. ; Goings, J. ; Peng, B. ; Petrone, A. ; Henderson, T. ; Ranasinghe, D. ; Zakrzewski, V. G. ; Gao, J. ; Rega, N. ; Zheng, G. ; Liang, W. ; Hada, M. ; Ehara, M. ; Toyota, K. ; Fukuda, R. ; Hasegawa, J. ; Ishida, M. ; Nakajima, T. ; Honda, Y. ; Kitao, O. ; Nakai, H. ; Vreven, T. ; Throssell, K. ; Montgomery, J. A. ; Peralta, J. E. ; Ogliaro, F. ; Bearpark, M. J. ; Heyd, J. J. ; Brothers, E. N. ; Kudin, K. N. ; Staroverov, V. N. ; Keith, T. A. ; Kobayashi, R. ; Normand, J. ; Raghavachari, K. ; Rendell, A. P. ; Burant, J. C. ; Iyengar, S. S. ; Tomasi, J. ; Cossi, M. ; Millam, J. M. ; Klene, M. ; Adamo, C. ; Cammi, R. ; Ochterski, J. W. ; Martin, R. L. ; Morokuma, K. ; Farkas, O. ; Foresman, J. B. ; Fox, D. J. Gaussian 16. Revision B.01; Gaussian Inc.: Wallingford CT, 2016.

[113] Scriven E., Powell B. J. (2009). Toward the parametrization
of the
Hubbard model for salts of bis­(ethylenedithio)­tetrathiafulvalene:
A density functional study of isolated molecules. J. Chem. Phys..

[114] Di Maiolo F., Masino M., Painelli A. (2017). Terahertz-pulse
driven
modulation of electronic spectra: Modeling electron-phonon coupling
in charge-transfer crystals. Phys. Rev. B.

