# Supplementary material for: Particle–Hole Symmetry Breaking in Nitrogen-Decorated Triphenylmethyl Radical Emitters
Source: J Phys Chem A. 2026 Jan 31;130(6):1338–50. doi: 10.1021/acs.jpca.5c08128 (PMC12908118; doi:10.1021/acs.jpca.5c08128)
Supplement: Supplementary file 1 [file jp5c08128_si_001.pdf]

Supplementary Information

Particle–Hole Symmetry Breaking in Nitrogen-Decorated Triphenylmethyl Radical Emitters

Alessio Graziano Rizzo, Marco Tommaso Barreca, and Francesco Di Maiolo*

Department of Chemistry, Life Science and Environmental Sustainability, Università di Parma, 43124 Parma, Italy.

E-mail: francesco.dimaiolo@unipr.it

Table of Contents

S1 *Ab initio* Computational Details **S3**

S2 QD-NEVPT2 Energies and CASSCF Molecular Orbitals **S4**

S3 Comparison of PPP-Idealized and DFT-Optimized Geometries **S12**

S4 The PPP Hartree-Fock Molecular Orbitals **S13**

S5 The Difference Operator **S20**

S6 Inductive effects in the trityl radical **S22**

S7 Particle-Hole Symmetry Breaking in Trityl-2N and Trityl-4N **S23**

S8 Additional Nitrogen-Decorated Trityl Radicals **S25**

S9 Cartesian coordinates **S31**

References **S40**

S1 *Ab initio* Computational Details

Geometry optimizations for the trityl radicals shown in Fig.1 in the main text were carried out at the DFT level using the UBHandHLYP functional[1,2] and the 6-31G(d,p) basis set in the gas phase. All structures were optimized in their doublet ground states. All geometry optimizations were carried out using the Gaussian16 software package.[3]

CASSCF calculations were carried out on the UBHandHLYP-optimized doublet ground-state geometries. To incorporate dynamic electron correlation, the CASSCF state energies were further refined using the van Vleck quasi-degenerate (QD) extension to strongly contracted second-order N-electron valence state perturbation theory (SC-NEVPT2).[4] All the CASSCF/QD-NEVPT2 calculations were done with the Orca package (version 6.0.1).[5]

CASSCF calculations were performed using (5,5) active space, with results reported in Section S2. All calculations employed the def2-SVP basis set, along with the Resolution of Identity (RI) approximation using the def2/JK auxiliary basis set. The first four doublet roots were computed in each case. The (5,5) active space includes five electrons in five frontier MOs. Second-order perturbative corrections were generally modest across all systems. The magnitude of the PT2 corrections to the CASSCF excitation energies varies across the trityl radical series. For the parent trityl radical, the PT2 contribution amounts to approximately 0.59 eV for the D_1 state and 0.52 eV for D_2. Upon introducing two aza nitrogens (trityl-2N), these corrections decrease markedly, dropping to 0.27 eV for D_1 and 0.16 eV for D_2. In trityl-4N, the PT2 shifts become 0.23 eV for D_1 and 0.68 eV for D_2. For trityl-6N, the lowest doubly degenerate excited doublet sate receives correlation correction of about 0.38 eV.

S2 QD-NEVPT2 Energies and CASSCF Molecular Orbitals

The CASSCF(5,5)/QD-NEVPT2 calculations yield transition energies of 2.975 eV, 2.977 eV, and 3.944 eV for the D_1, D_2, and D_3 states of the trityl radical, respectively. In comparison, the PPP-RASCI(h,p,hp) approach, employing a RAS2 active space of 5 electrons in 5 MOs, reproduces a perfect double degeneracy of D_1 and D_2 at 2.869 eV, and a higher-lying D_3 state at 3.826 eV. The CASSCF frontier MOs of trityl obtained with the (5,5) active space are shown in Fig.S1. QD-NEVPT2 transition energies for the aza nitrogen-decorated trityl radicals are reported in Tab.S1. Relevant frontier MOs obtained with the (5,5) active space are shown in Figs. S2, S3, S4, S5, S6, and S7, respectively.

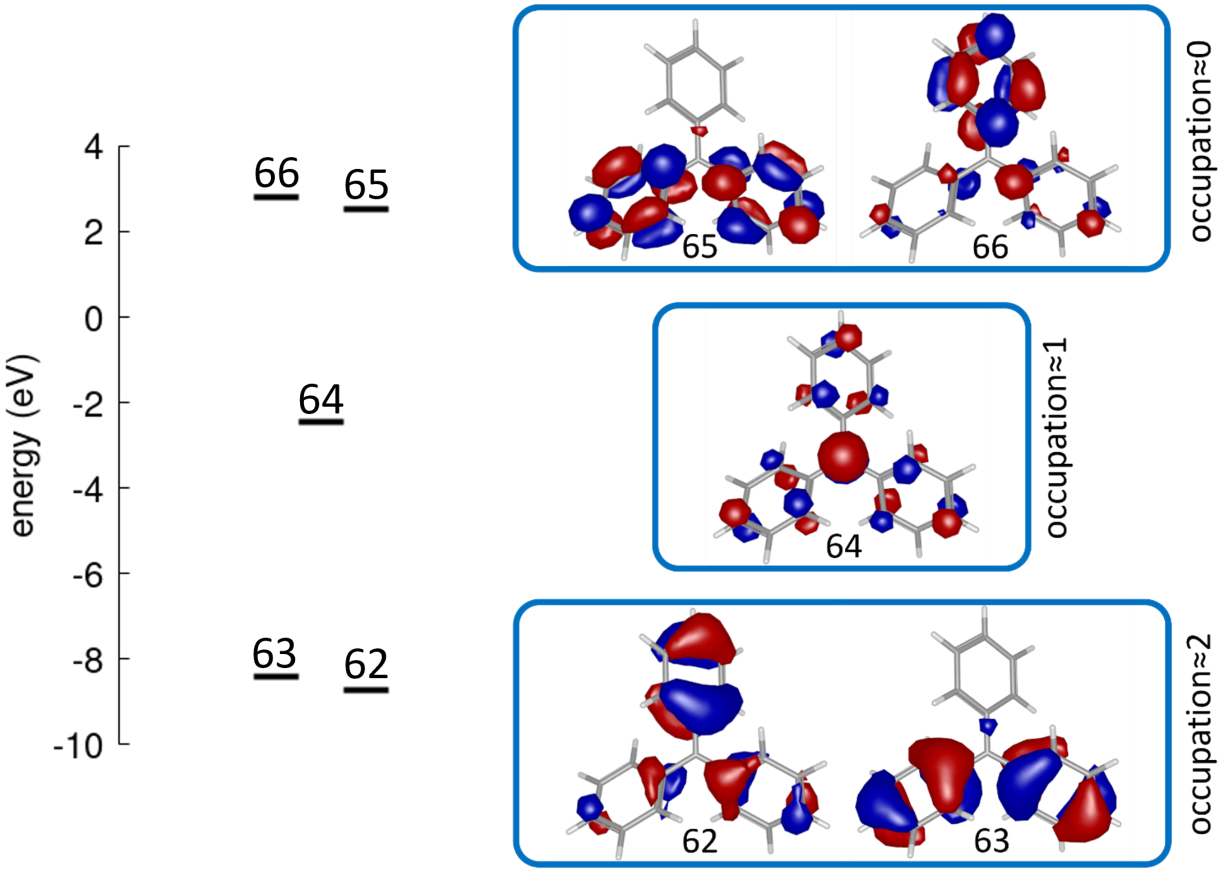

Figure S1: CASSCF frontier molecular orbitals of trityl calculated with (5,5) active space using the def2-SVP basis set.

Table S1: Transition energies (in eV) for the first three excited doublet states of trityl-1N, trityl-2N, trityl-3N, trityl-4N, trityl-5N, and trityl-6N, computed at the PPP-RASCI(h,p,hp) and CASSCF(5,5)/QD-NEVPT2 levels of theory. Corresponding oscillator strengths are given in parentheses.

	PPP-RASCI(h,p,hp)	CASSCF(5,5)/QD-NEVPT2
	**trityl-1N**	
$E(D_1)$	2.856 (0.0000)	3.124 (0.0121)
$E(D_2)$	2.872 (0.0001)	3.276 (0.3079)
$E(D_3)$	3.773 (0.0275)	4.009 (0.6989)
	**trityl-2N**	
$E(D_1)$	2.853 (0.0000)	3.088 (0.0007)
$E(D_2)$	2.870 (0.0002)	3.236 (0.0258)
$E(D_3)$	3.791 (0.0000)	4.010 (0.7881)
	**trityl-3N**	
$E(D_1)$	2.849 (0.0000)	3.101 (0.0122)
$E(D_2)$	2.864 (0.0006)	3.264 (0.0231)
$E(D_3)$	3.802 (0.0014)	3.999 (0.7605)
	**trityl-4N**	
$E(D_1)$	2.848 (0.0003)	3.074 (0.0015)
$E(D_2)$	2.863 (0.0005)	3.177 (0.1025)
$E(D_3)$	3.825 (0.0048)	3.954 (0.7044)
	**trityl-5N**	
$E(D_1)$	2.849 (0.0008)	3.212 (0.0204)
$E(D_2)$	2.852 (0.0006)	3.244 (0.0051)
$E(D_3)$	3.825 (0.0448)	4.201 (0.5526)
	**trityl-6N**	
$E(D_1)$	2.850 (0.0008)	3.171 (0.0209)
$E(D_2)$	2.850 (0.0008)	3.188 (0.0564)
$E(D_3)$	3.868 (0.0000)	4.194 (0.7308)

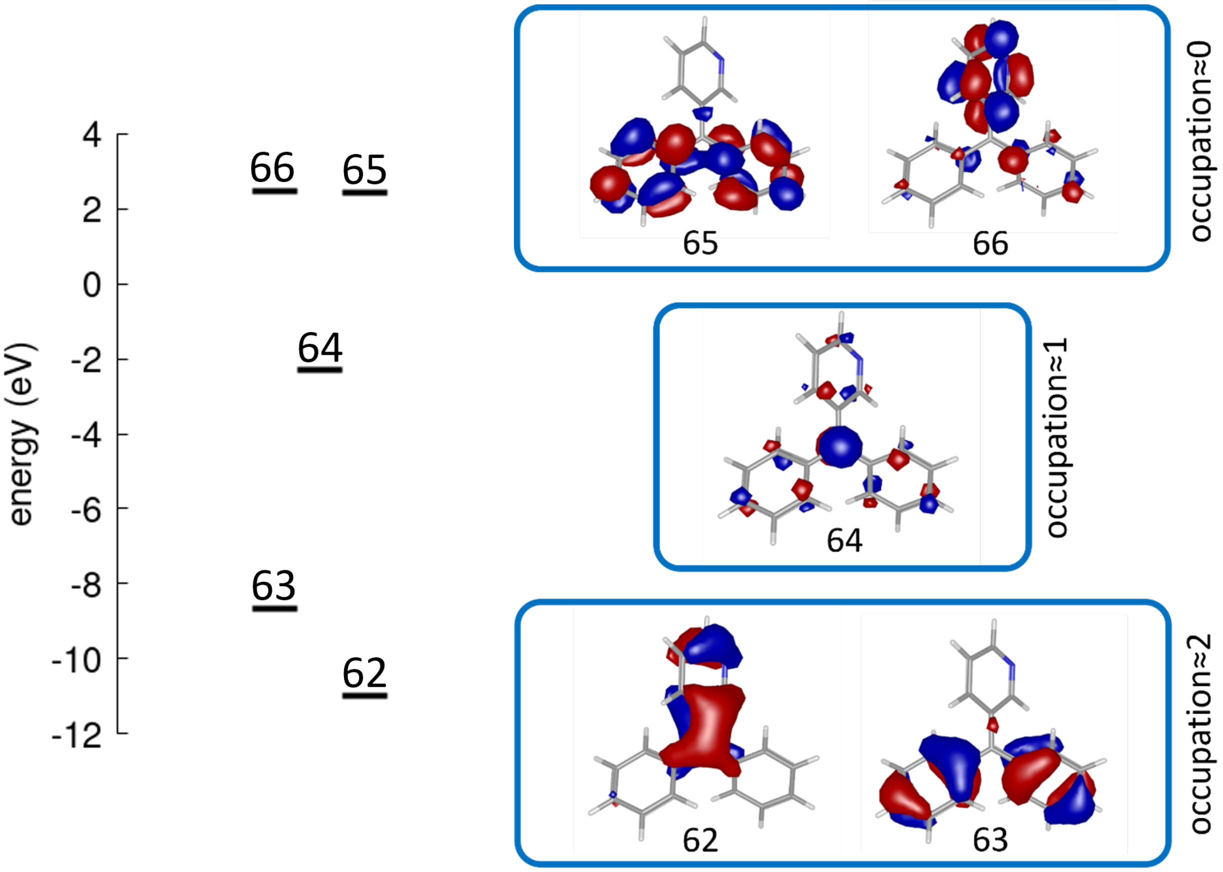

Figure S2: CASSCF frontier molecular orbitals of trityl-1N calculated with (5,5) active space using the def2-SVP basis set.

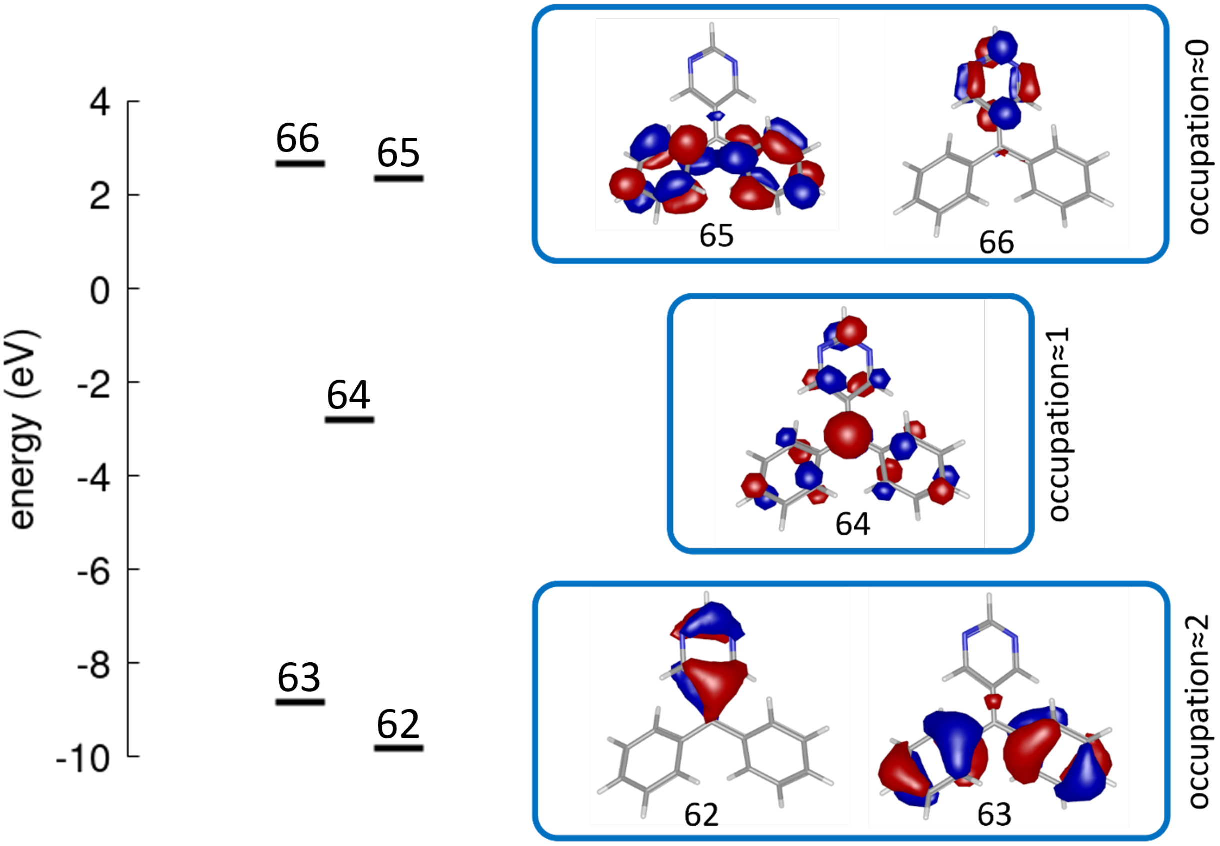

Figure S3: CASSCF frontier molecular orbitals of trityl-2N calculated with (5,5) active space using the def2-SVP basis set.

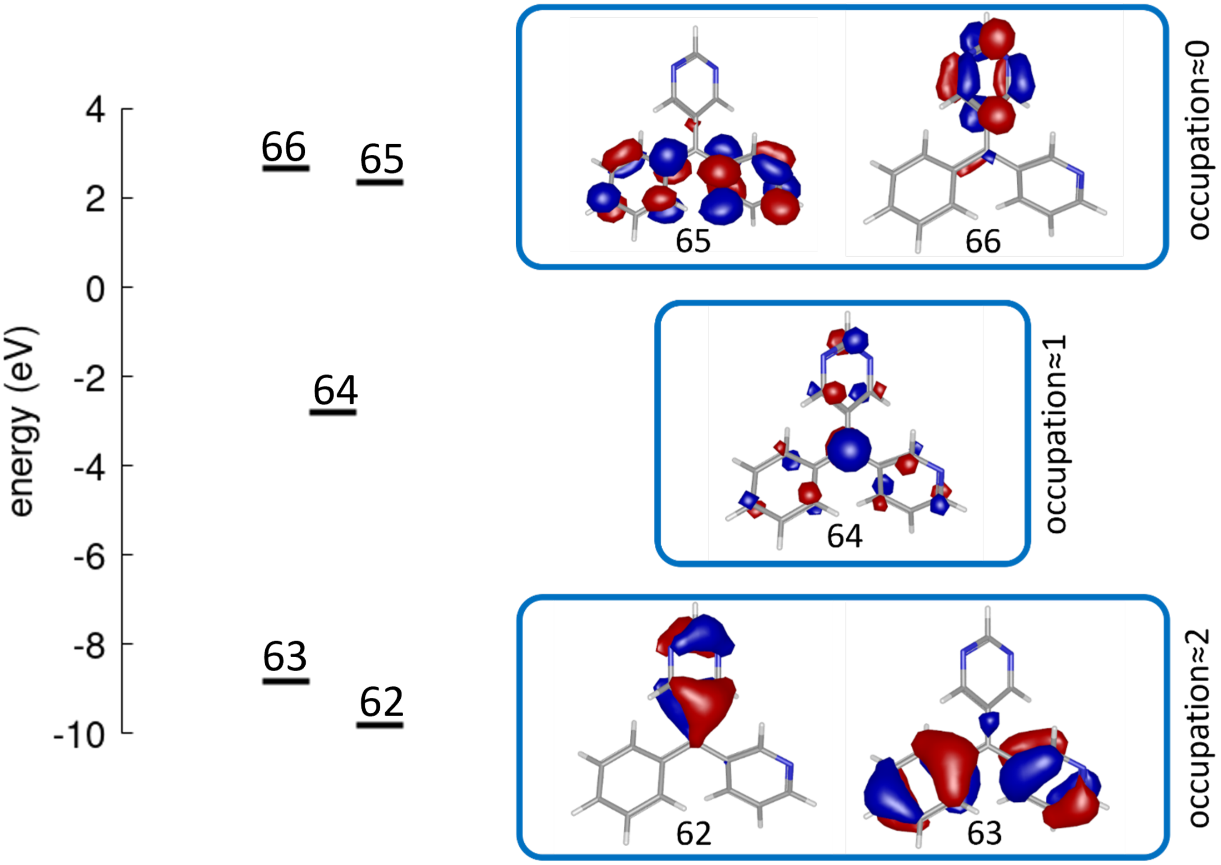

Figure S4: CASSCF frontier molecular orbitals of trityl-3N calculated with (5,5) active space using the def2-SVP basis set.

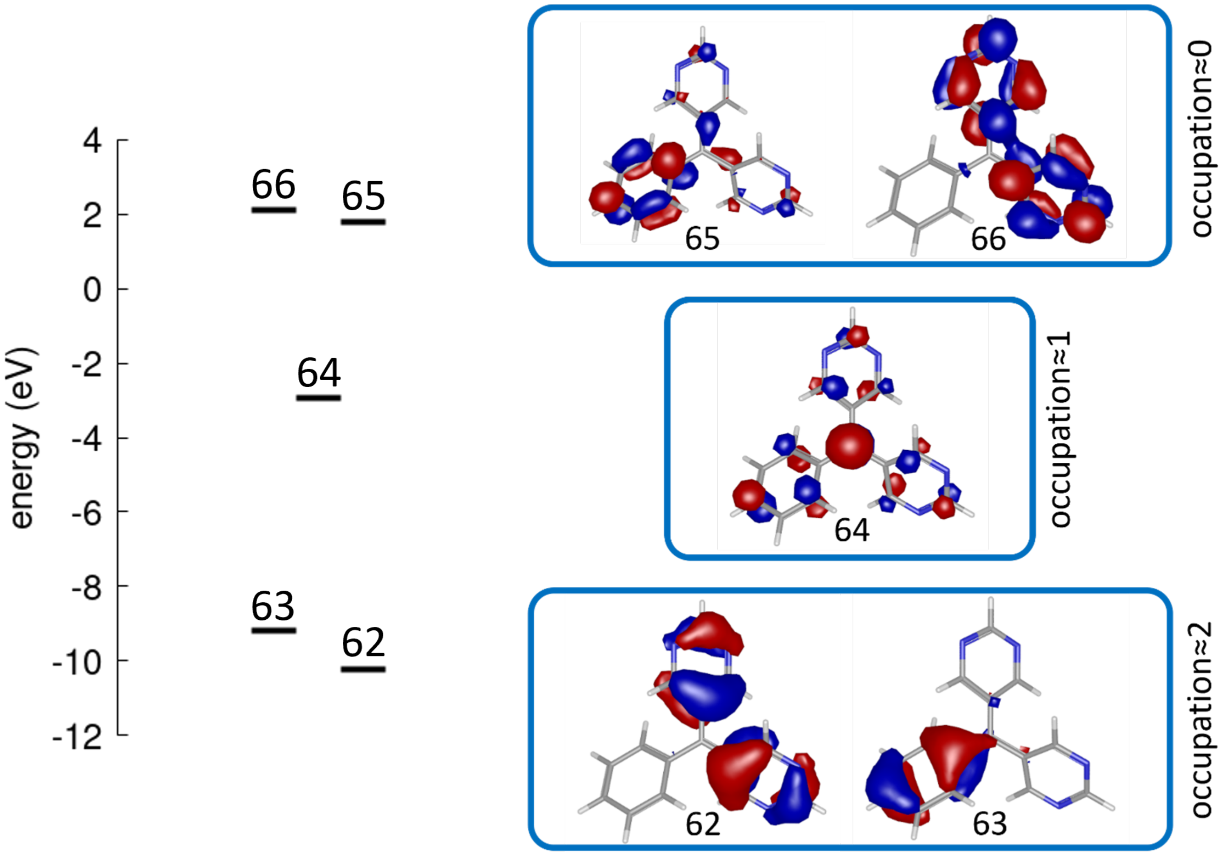

Figure S5: CASSCF frontier molecular orbitals of trityl-4N calculated with (5,5) active space using the def2-SVP basis set.

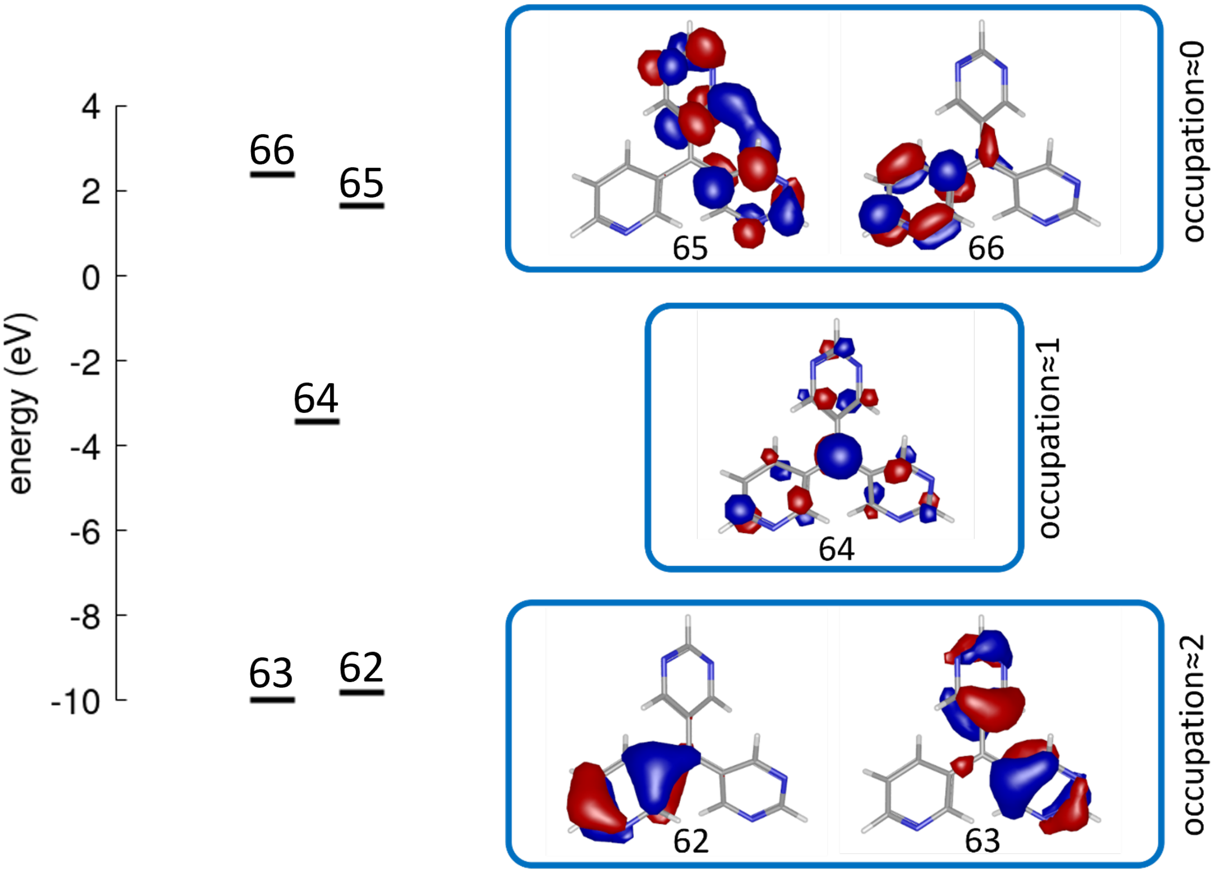

Figure S6: CASSCF frontier molecular orbitals of trityl-5N calculated with (5,5) active space using the def2-SVP basis set.

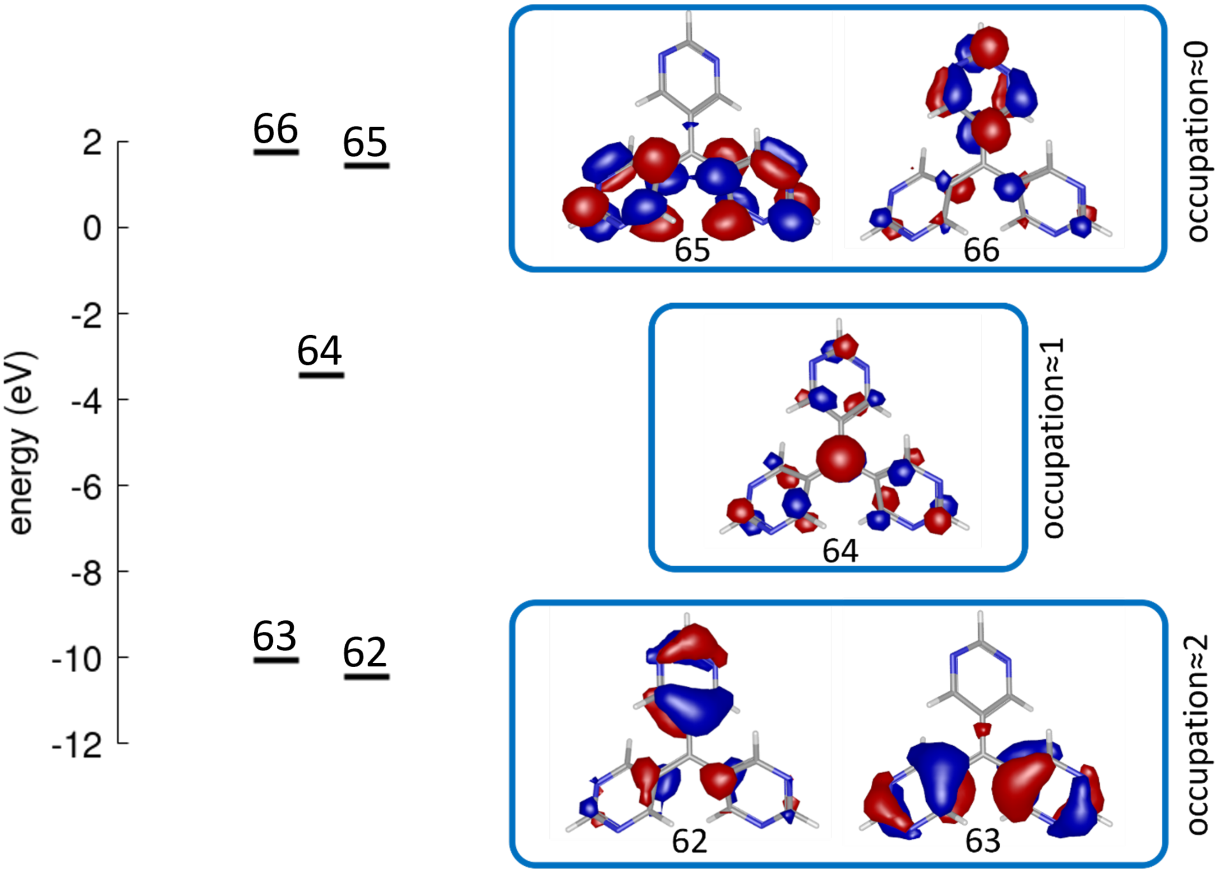

Figure S7: CASSCF frontier molecular orbitals of trityl-6N calculated with (5,5) active space using the def2-SVP basis set.

S3 Comparison of PPP-Idealized and DFT-Optimized Geometries

Table S2 summarizes the deviations in heavy-atom bond lengths and angles between the DFT-optimized and PPP-idealized structures. In all cases, bond length deviations are modest, typically within 0.03-0.06 Å on average, with maxima below 0.081 Å, and bond angle deviations are limited to a few degrees, with RMS values of ∼1°-4°.

Table S2: Deviations between DFT-optimized (UBHandHLYP/6-31G(d,p)) heavy-atom geometries and the idealized PPP geometries (all bonds 1.40 Å, all angles 120°; hydrogens omitted).

System	Bond lengths vs 1.40 Å		Bond angles vs 120°	
	RMSD (Å)	Max $\|\Delta r\|$ (Å)	RMSD (°)	Max $\|\Delta\alpha\|$ (°)
trityl	0.025	0.059	1.27	2.67
trityl-1N	0.034	0.079	1.81	4.64
trityl-2N	0.041	0.079	2.47	6.35
trityl-3N	0.047	0.080	2.78	6.35
trityl-4N	0.052	0.080	3.23	6.34
trityl-5N	0.057	0.080	3.47	6.33
trityl-6N	0.062	0.081	3.83	6.31

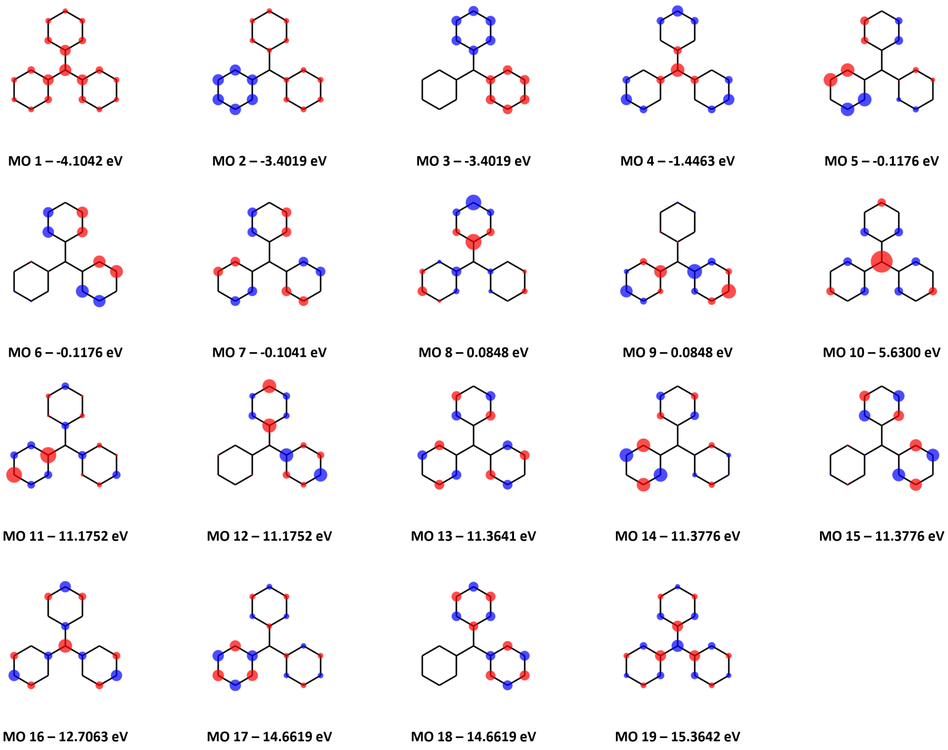

Figure S8: Energy levels of the trityl radical computed at the PPP–HF level, along with the corresponding HF molecular orbitals. MOs 8 and 9 form the doubly degenerate HOMO; MO 10 is the SOMO; and MOs 11 and 12 constitute the doubly degenerate LUMO. The same PPP model parameters as those used in the main text were employed.

S4 The PPP Hartree-Fock Molecular Orbitals

The operators $\hat{b}_{k\sigma}^{(\dagger)}$ that annihilate (create) an electron with spin σ in the k-th molecular orbital (MO) can be written as linear combination of the $\hat{a}_{\mu\sigma}^{(\dagger)}$ (on-site) operators:

$$\hat{b}_{k\sigma} = \sum_{\mu} c_{\mu,k}\hat{a}_{\mu\sigma} \tag{S1}$$

$$\hat{b}_{k\sigma}^{\dagger} = \sum_{\mu} c_{\mu,k}\hat{a}_{\mu\sigma}^{\dagger} \tag{S2}$$

where the expansion coefficients are obtained upon diagonalization of the Fock operator in Eq. 4, main text. The full sets of PPP-HF molecular orbitals for the trityl radicals in Fig.5 main text are shown in Figs. S8, S9, S10, S11, S12, S13, and S14.

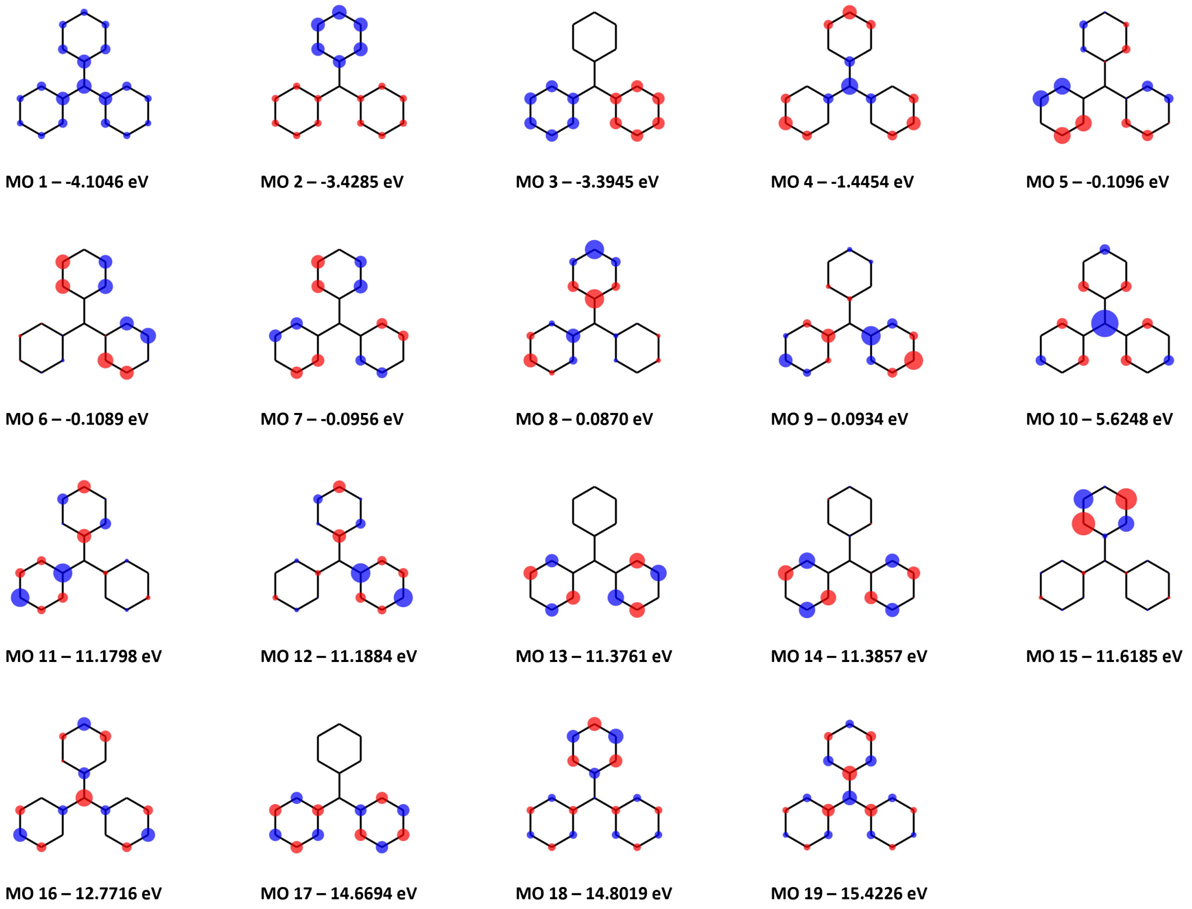

Figure S9: Energy levels of trityl-1N computed at the PPP–HF level, together with the corresponding HF molecular orbitals. The nitrogen atom occupies one meta position of the top phenyl ring; MO 9 is the HOMO, MO 10 is the SOMO, and MO 11 is the LUMO. The PPP model parameters are the same as those used in the main text.

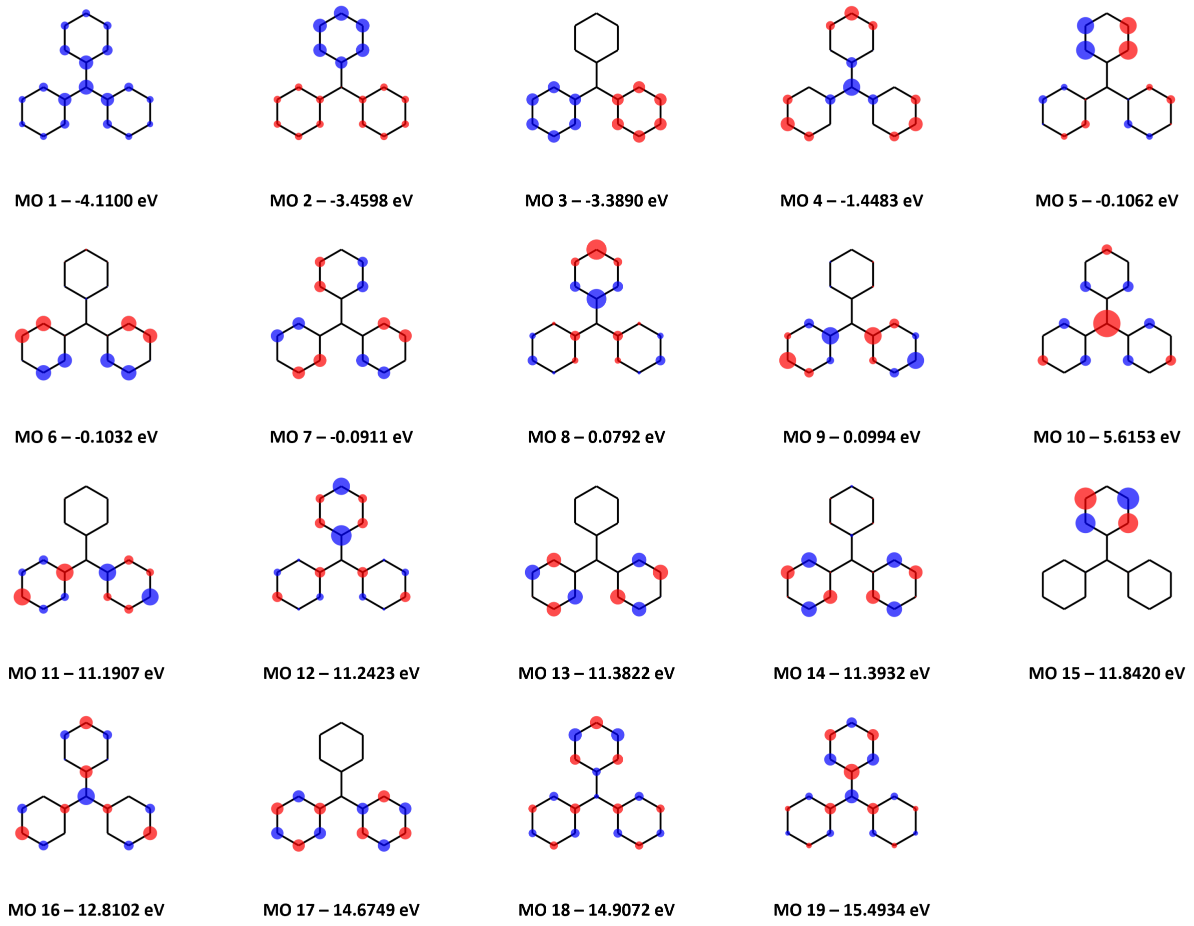

Figure S10: Energy levels of trityl-2N computed at the PPP–HF level, together with the corresponding HF molecular orbitals. The nitrogen atoms occupy the two meta positions of the top phenyl ring; MO 9 is the HOMO, MO 10 is the SOMO, and MO 11 is the LUMO. The PPP model parameters are the same as those used in the main text.

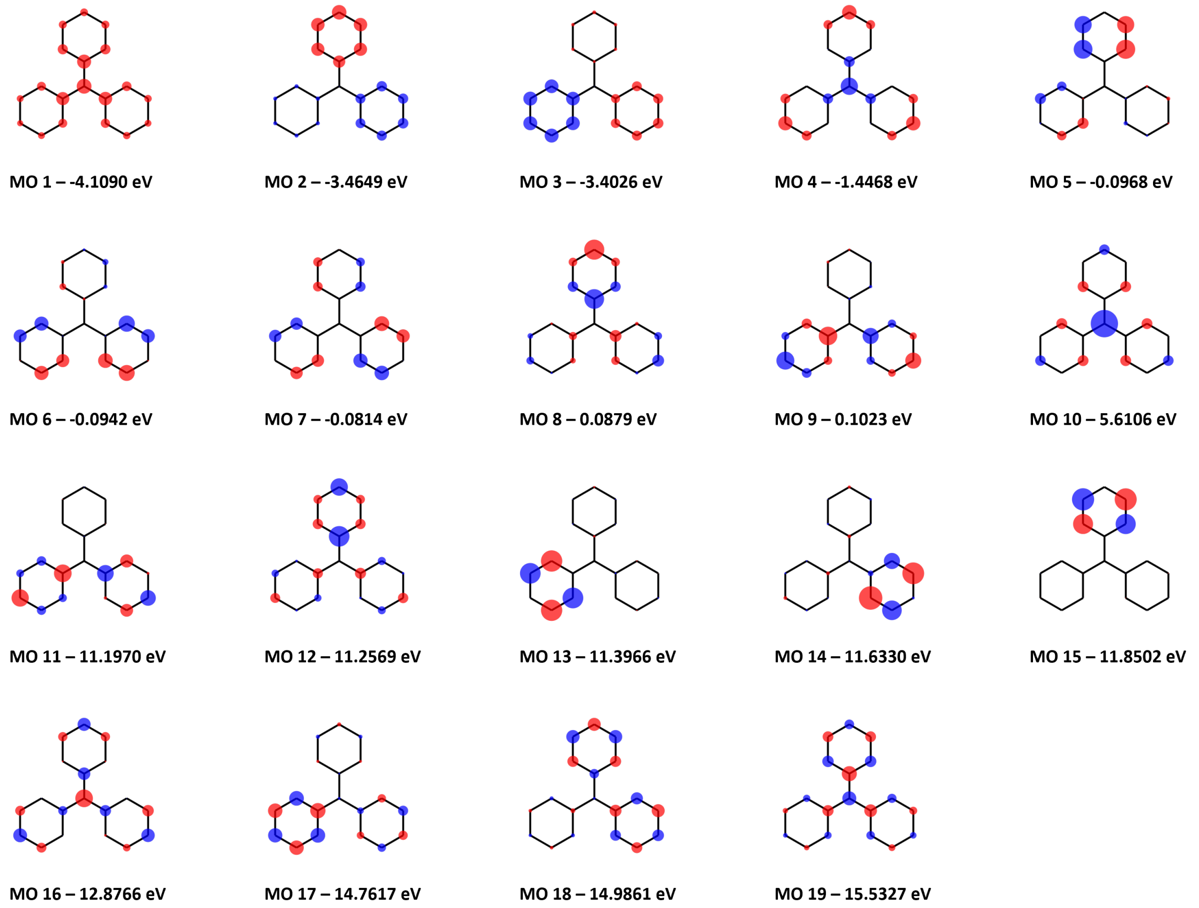

Figure S11: Energy levels of trityl-3N computed at the PPP–HF level, together with the corresponding HF molecular orbitals. The nitrogen atoms occupy both meta positions of the top phenyl ring and one meta position of the bottom-right phenyl ring. MO 9 is the HOMO, MO 10 is the SOMO, and MO 11 is the LUMO. The PPP model parameters are the same as those used in the main text.

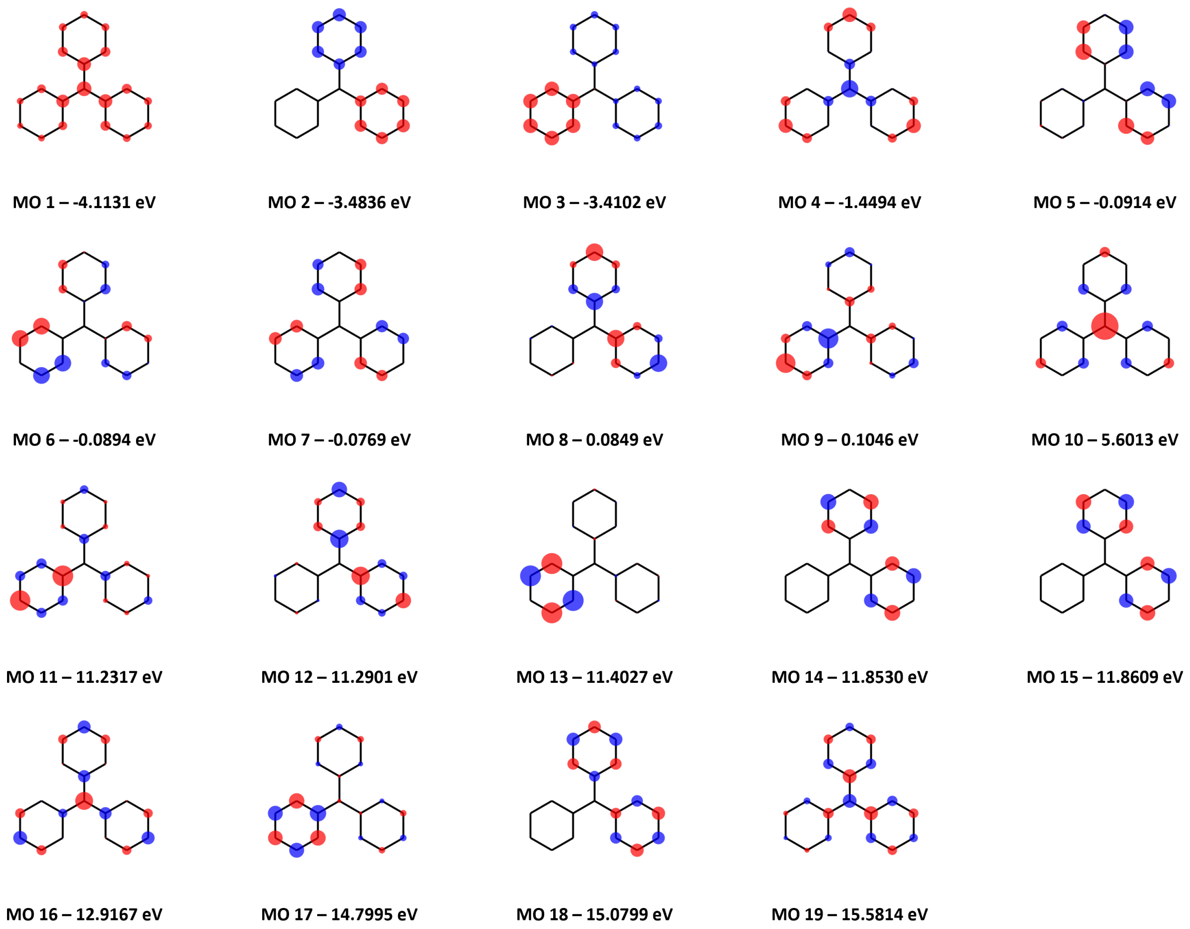

Figure S12: Energy levels of trityl-4N computed at the PPP–HF level, together with the corresponding HF molecular orbitals. The nitrogen atoms are positioned at the meta sites of the top and bottom-right phenyl rings; MO 9 is the HOMO, MO 10 is the SOMO, and MO 11 is the LUMO. The PPP model parameters are identical to those used in the main text.

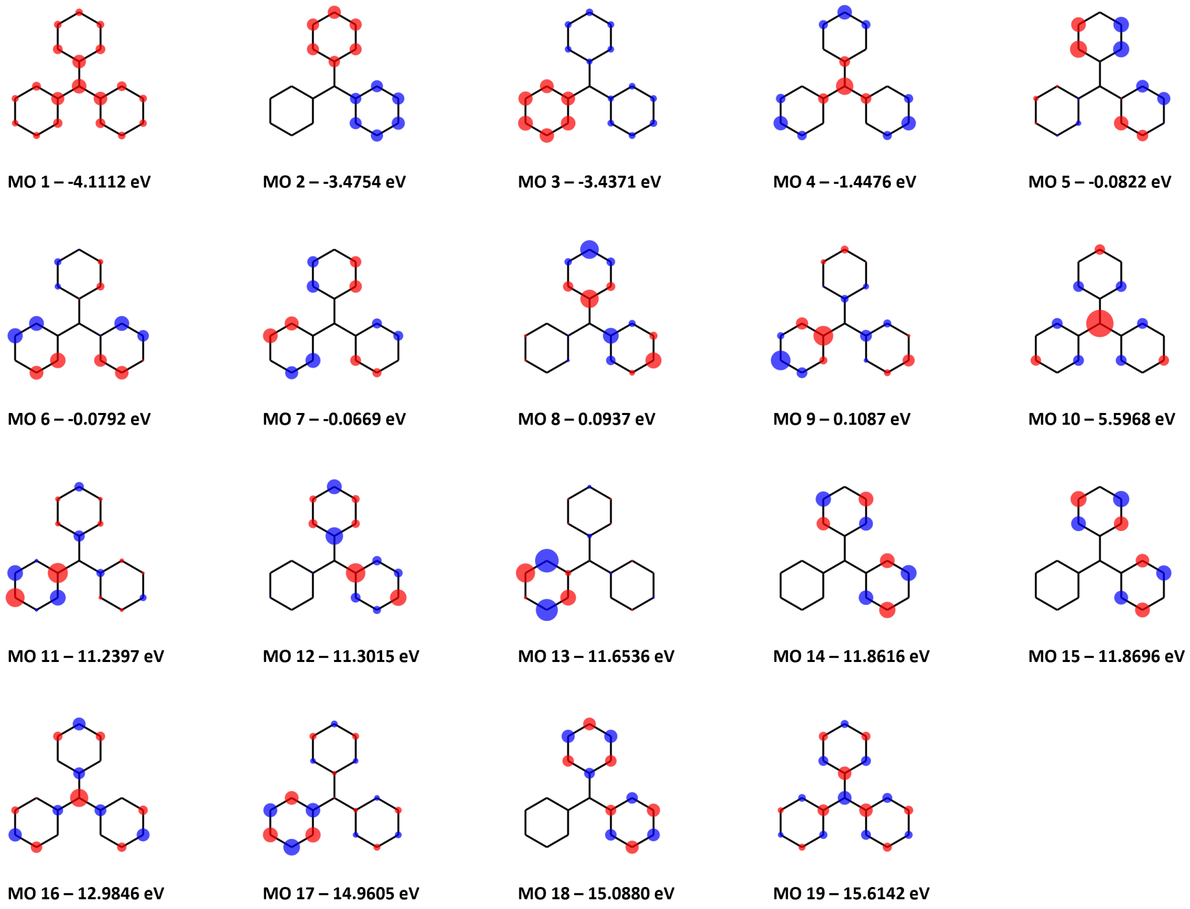

Figure S13: Energy levels of trityl-5N computed at the PPP–HF level, together with the corresponding HF molecular orbitals. The nitrogen atoms occupy the meta positions of the top and bottom-right phenyl rings and one meta position of the bottom-left phenyl ring. MO 9 is the HOMO, MO 10 is the SOMO, and MO 11 is the LUMO. The PPP model parameters reported in the main text.

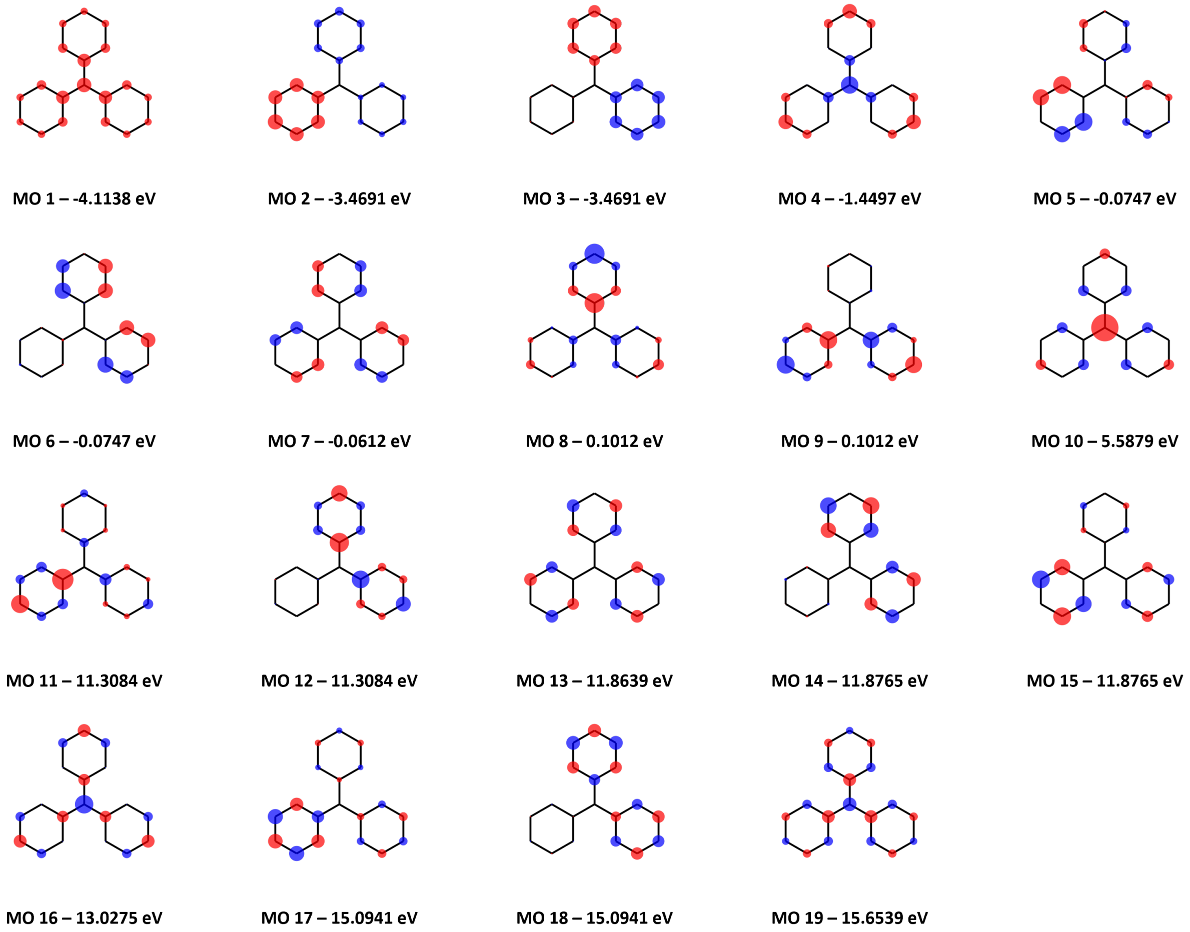

Figure S14: Energy levels of trityl-6N computed at the PPP–HF level, together with the corresponding HF molecular orbitals. The nitrogen atoms occupy the meta positions of all three phenyl rings; MOs 8 and 9 form the doubly degenerate HOMO, MO 10 is the SOMO, and MOs 11 and 12 constitute the doubly degenerate LUMO. The PPP model parameters reported in the main text.

S5 The Difference Operator

The operator $\hat{a}^{\dagger}_{\mu\sigma}$ creates one electron, while $\hat{a}_{\mu\sigma}$ destroys one electron. This is equivalent to saying that the annihilation operator $\hat{a}_{\mu\sigma}$ creates a hole, whereas $\hat{a}^{\dagger}_{\mu\sigma}$ annihilates a hole.[6] Mathematically, we have the following particle-hole transformation (pht):

$$\hat{c}^{\dagger}_{\mu\sigma} = \hat{a}_{\mu\sigma} \tag{S3}$$

$$\hat{c}_{\mu\sigma} = \hat{a}^{\dagger}_{\mu\sigma} \tag{S4}$$

The operator $\hat{c}^{(\dagger)}_{\mu\sigma}$ annihilates (creates) a hole with spin σ in the atomic orbital on site μ. Since the commutation properties of the hole operators are identical to those of the particle operators, the real-space PPP Hamiltonian in Eq.1 main text in terms of hole operators reads:

$$\begin{aligned}\hat{H}^{pht}_{PPP} &= \sum_{\mu} \varepsilon_{\mu} \hat{N}_{\mu} - \sum_{\mu\nu,\nu>\mu} \sum_{\sigma} t_{\mu\nu} \left(\hat{c}^{\dagger}_{\mu\sigma} \hat{c}_{\nu\sigma} + \hat{c}^{\dagger}_{\nu\sigma} \hat{c}_{\mu\sigma} \right) \\ &\quad + \sum_{\mu} U_{\mu} \hat{N}_{\mu\uparrow} \hat{N}_{\mu\downarrow} + \sum_{\mu\nu,\nu>\mu} V_{\mu\nu} \left(Z_{\mu} - \hat{N}_{\mu} \right) \left(Z_{\nu} - \hat{N}_{\nu} \right) && \text{(S5)} \\ &\quad + \sum_{\mu} 2\varepsilon_{\mu} \left(1 - \hat{N}_{\mu} \right) + \sum_{\mu} U_{\mu} \left(1 - \hat{N}_{\mu} \right) && \text{(S6)}\end{aligned}$$

where $\hat{N}_{\mu} = \sum_{\sigma} \hat{c}^{\dagger}_{\mu\sigma} \hat{c}_{\mu\sigma}$ counts the total number of holes on site μ. The first two lines in the above equation have the same form as in the original PPP hamiltonian in Eq.1 main text, whereas the last line is different. Accordingly, the last line represents the difference between the original and the ph-transformed hamiltonian:

$$\hat{D} = \sum_{\mu} \left[U_{\mu} + 2\varepsilon_{\mu} \right] \left(1 - \hat{N}_{\mu} \right) \tag{S7}$$

Going back to the particle operators, we get:

$$\hat{D} = \sum_{\mu} [U_\mu + 2\varepsilon_\mu] (\hat{n}_\mu - 1) \quad \text{(S8)}$$

that is the expression in Eq.10 main text. In the MO basis, the difference operator reads:

$$\hat{D} = \sum_{ij} \sum_{\sigma} \left[\sum_{\mu} (U_\mu + 2\varepsilon_\mu) (c_{i\mu} c_{j\mu} - \delta_{ij}) \right] \hat{b}^\dagger_{i\sigma} \hat{b}_{j\sigma} \quad \text{(S9)}$$

where δ_{ij} is the Kronecker delta and $c_{i\mu}$ are the expansion coefficients of the i-th MO on the AOs.

S6 Inductive effects in the trityl radical

In the PPP framework, the particle–hole symmetry of alternant hydrocarbons can be altered by introducing inductive effects, which manifest through variations in the on-site energies.[7] Within the trityl skeleton, tertiary carbon atoms (such as C6, C7, C8, and C14 in Fig. S15, panel a) possess slightly stronger electron-donating character than secondary carbons. This electronic imbalance can be represented by assigning small positive on-site energy to these tertiary centers. Figure S15 reports the results obtained when the on-site energies of the four tertiary carbons are set to 0.05, 0.15, and 0.25 eV. These perturbations have a negligible influence on the transition energies of the lowest doubly degenerate doublet state (panel a), yet they slightly enhance the oscillator strength (panel c) as particle–hole symmetry becomes progressively broken by the nonequivalent carbon environments in trityl (panel b).

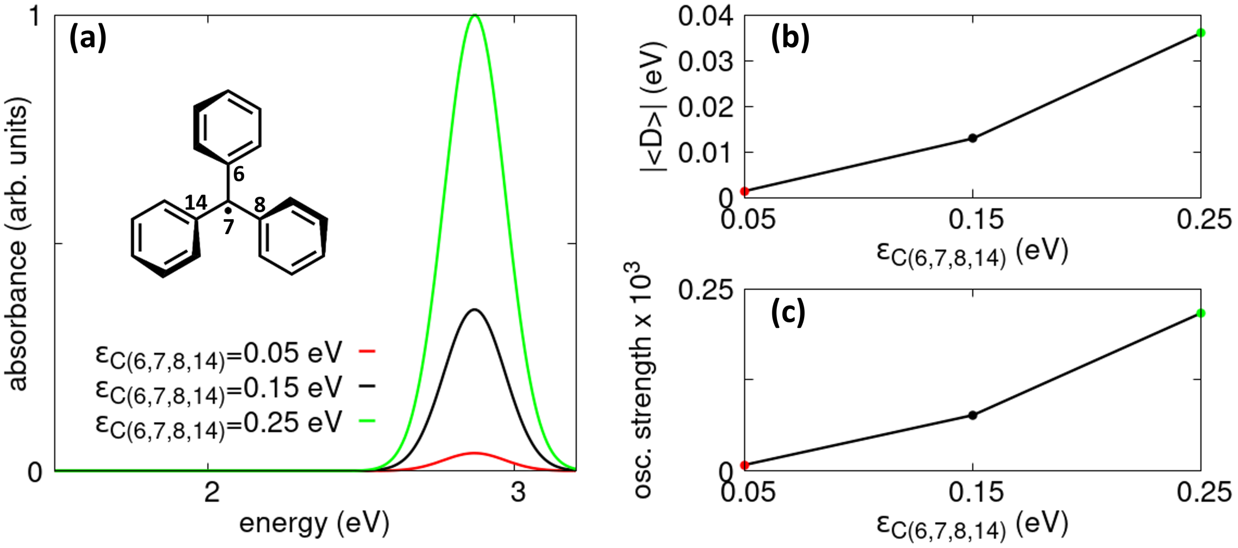

Figure S15: Absorption spectra (panel a), expectation values of the difference operator (panel b), and oscillator strengths associated with the first doubly degenerate doublet state (panel c) of the trityl radical. Calculations were performed at the PPP-RASCI(h,p,hp) level by assigning PPP on-site energies of 0.05 eV (red), 0.15 eV (black), and 0.25 eV (green) to carbons 6, 7, 8, and 14. All other model parameters are identical to those used in Fig. 2 of the main text.

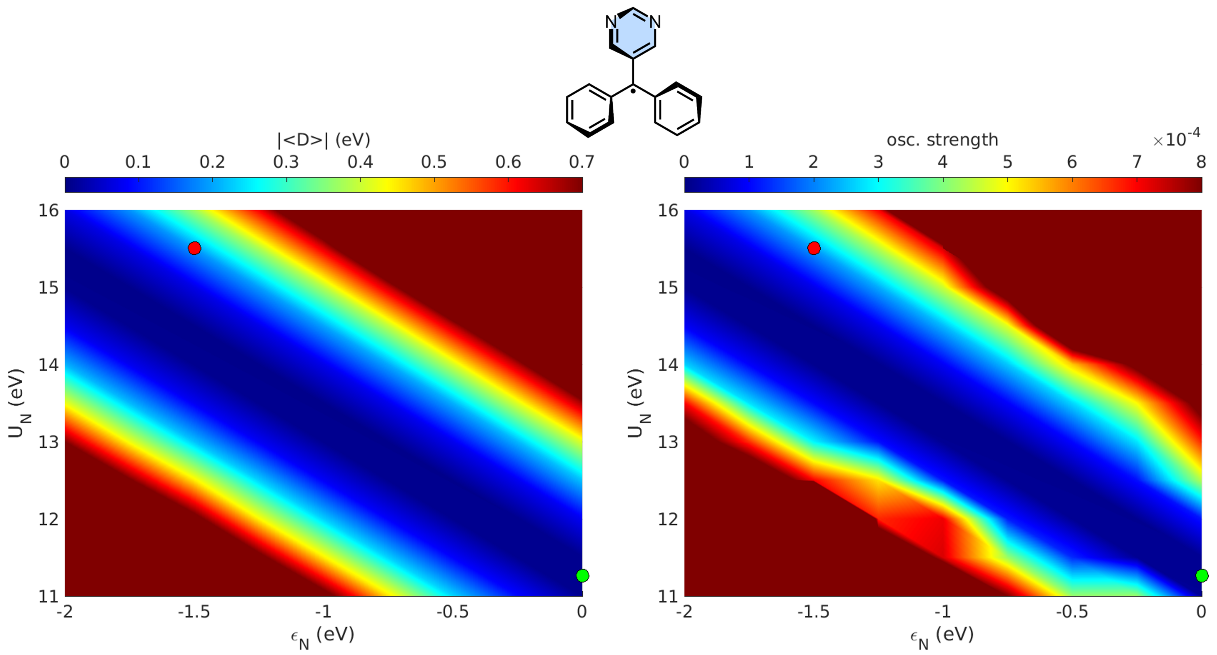

Figure S16: Particle-Hole Symmetry Breaking in trityl-2N. Left color map illustrates how $|\langle \hat{D} \rangle|$ varies with U_N and ε_N. Right color map reports how the oscillator strength of the first absorption band depends on U_N and ε_N. The green dot indicates the parameters relevant to trityl, while the red dot those relevant to trityl-2N. Calculations performed at the PPP-RASCI(h,p,hp) level, using the model parameters specified in the main text.

S7 Particle-Hole Symmetry Breaking in Trityl-2N and Trityl-4N

Similarly to Fig.4 main text, in Figs. S16 and S17, we report $|\langle \hat{D} \rangle|$ and the oscillator strength dependence on the meta positions in the phenyl rings parameters, specifically ε_N and U_N. The green dot on both maps corresponds to the trityl parameters. Red dot corresponds to trityl-2N and trityl-4N parameters.

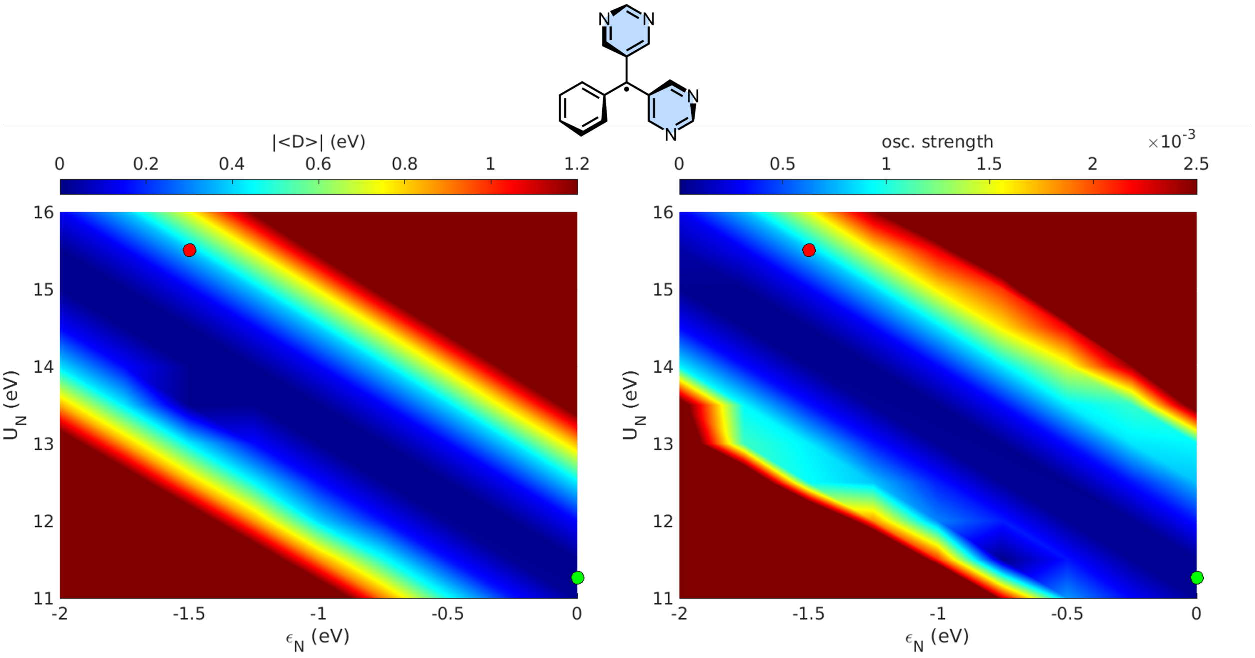

Figure S17: Particle-Hole Symmetry Breaking in trityl-4N. Left color map illustrates how $|\langle\hat{D}\rangle|$ varies with U_N and ε_N. Right color map reports how the oscillator strength of the first absorption band depends on U_N and ε_N. The green dot indicates the parameters relevant to trityl, while the red dot those relevant to trityl-4N. Calculations performed at the PPP-RASCI(h,p,hp) level, using the model parameters specified in the main text.

S8 Additional Nitrogen-Decorated Trityl Radicals

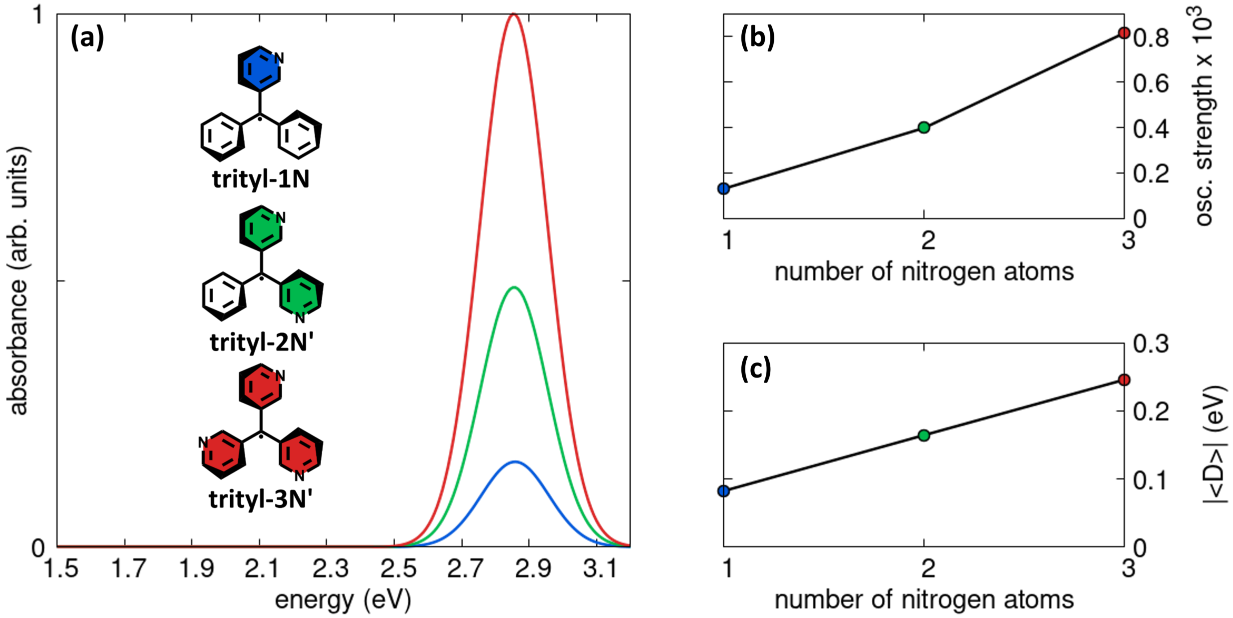

Figure S18: Effect of progressively introducing aza-nitrogen atoms at one meta position of the three phenyl rings of the trityl radical. Panel (a) shows the calculated absorption spectra for trityl-1N, trityl-2N', and trityl-3N'. Panel (b) reports the oscillator strength integrated over the 0–3.2 eV energy window, while panel (c) displays the absolute value of the ground state expectation value of the difference operator for the three trityl derivatives. In panel (a), all spectra are normalized to the maximum absorbance of the most intense curve. Model parameters are provided in the main text. All calculations were carried out at the PPP-RASCI(h,p,hp) level.

To examine the effect of increasing the number of nitrogen atoms when they are distributed across different phenyl rings, we performed PPP-RASCI(h,p,hp) calculations on two additional nitrogen-decorated trityl radicals denoted as trityl-2N' and trityl-3N', and compared them with trityl-1N, which corresponds to the structure already discussed in the main text. In trityl-2N', two nitrogen atoms occupy meta positions on two different phenyl rings, while in trityl-3N' one nitrogen atom is placed on each of the three phenyl rings. Despite this alternative distribution of nitrogen atoms, the main absorption band remains essentially unchanged at approximately 2.86 eV for trityl-1N, trityl-2N', and trityl-3N' (Fig. S18a). The absolute value of the ground state expectation value of the difference operator, $|\langle \hat{D} \rangle|$, increases monotonically from 0.08 eV in trityl-1N to 0.16 eV in trityl-2N' and to 0.25 eV in

trityl-3N' (panel c), indicating an almost additive contribution of approximately 0.08 eV per nitrogen atom when dopants are distributed over different rings. This contribution is slightly smaller than the ∼0.11 eV increase observed when nitrogen atoms are introduced pairwise on the same phenyl ring. The oscillator strength integrated over the lowest doublet excitations D_1 and D_2 – which are separated by approximately 0.02 eV in trityl-1N and trityl-2N' and become degenerate in trityl-3N' – increases with the number of nitrogen atoms. The corresponding PPP-HF molecular orbitals are shown in Figs. S19 and S20, while QD-NEVPT2 transition energies and the relevant CASSCF(5,5) orbitals are reported in Table S3 and Figs. S21 and S22.

Table S3: Transition energies (in eV) of the first three excited doublet states of trityl-2N' and trityl-3N', obtained at the PPP-RASCI(h,p,hp) and CASSCF(5,5)/QD-NEVPT2 levels of theory. The corresponding oscillator strengths are reported in parentheses.

	PPP-RASCI(h,p,hp)	CASSCF(5,5)/QD-NEVPT2
	**trityl-2N'**	
$E(D_1)$	2.851 (0.0003)	3.074 (0.0228)
$E(D_2)$	2.866 (0.0001)	3.189 (0.0053)
$E(D_3)$	3.765 (0.0409)	3.996 (0.7489)
	**trityl-3N'**	
$E(D_1)$	2.854 (0.0004)	3.102 (0.0163)
$E(D_2)$	2.854 (0.0004)	3.200 (0.0076)
$E(D_3)$	3.772 (0.0432)	4.056 (0.7355)

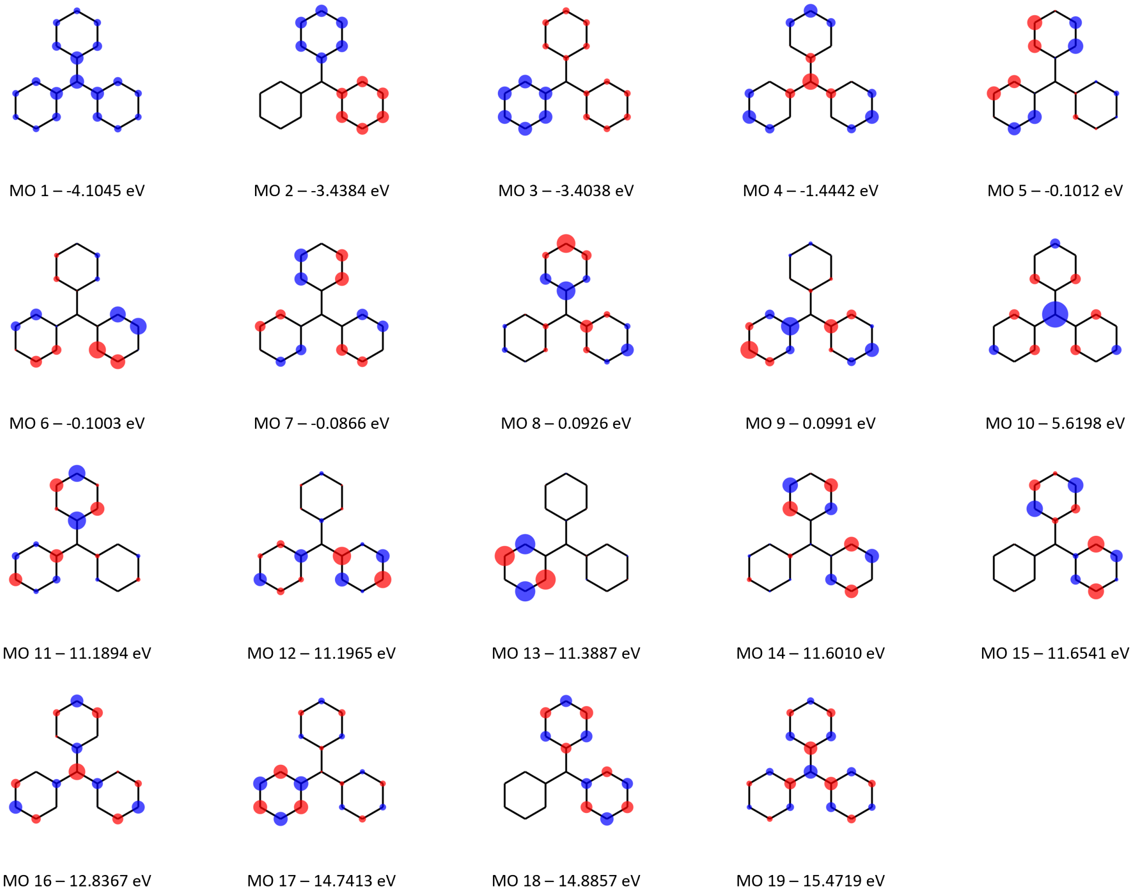

Figure S19: Energy levels of trityl-2N' obtained at the PPP–HF level, together with the corresponding HF molecular orbitals. One nitrogen atom occupies a meta position in the top phenyl ring, while the second nitrogen atom occupies a meta position in the bottom-right phenyl ring; MO 9 is the HOMO, MO 10 is the SOMO, and MO 11 is the LUMO. The PPP model parameters are the same as those used in the main text.

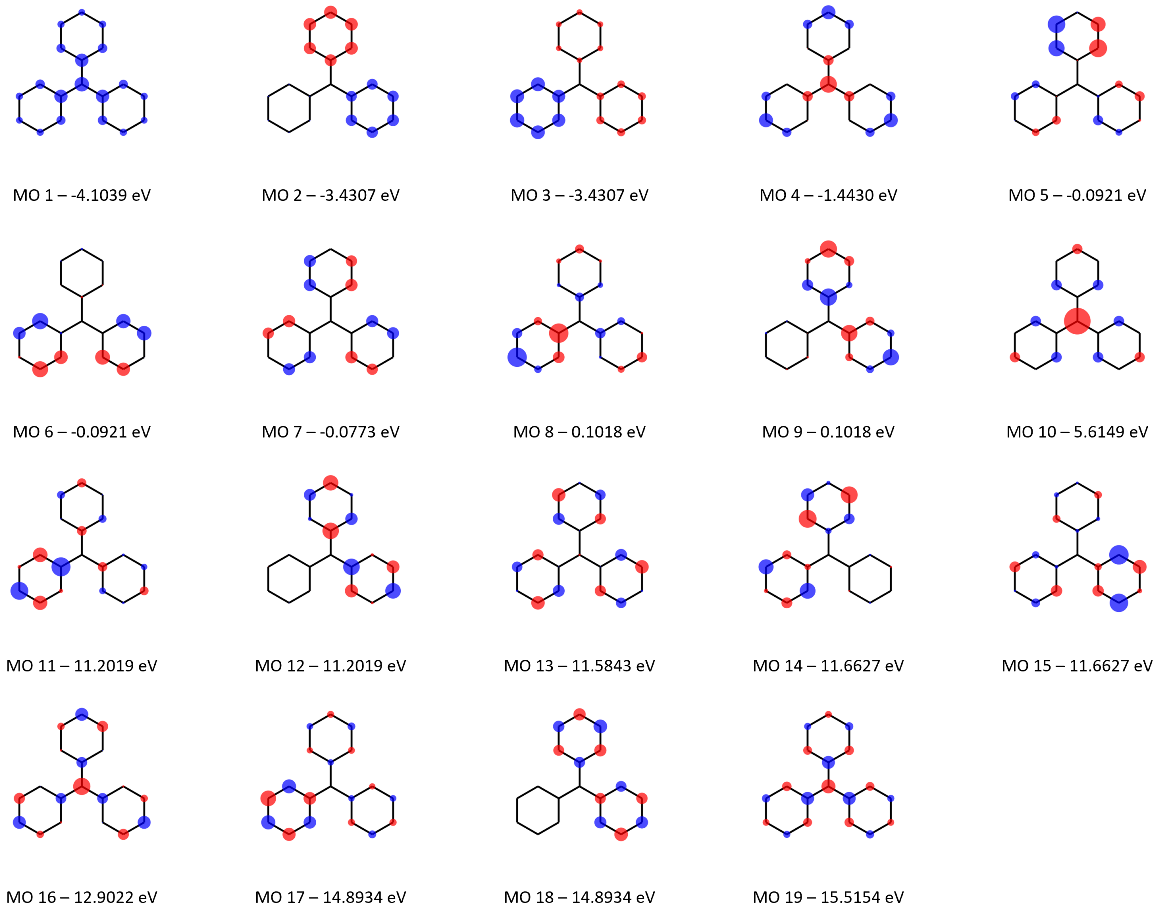

Figure S20: Energy levels of trityl-3N' calculated at the PPP–HF level, together with the corresponding HF molecular orbitals. One nitrogen atom occupies a meta position in the top phenyl ring, while the second and third nitrogen atoms occupy meta positions in the bottom-right and bottom-left phenyl rings, respectively; MO 9 is the HOMO, MO 10 is the SOMO, and MO 11 is the LUMO. The PPP model parameters are the same as those used in the main text.

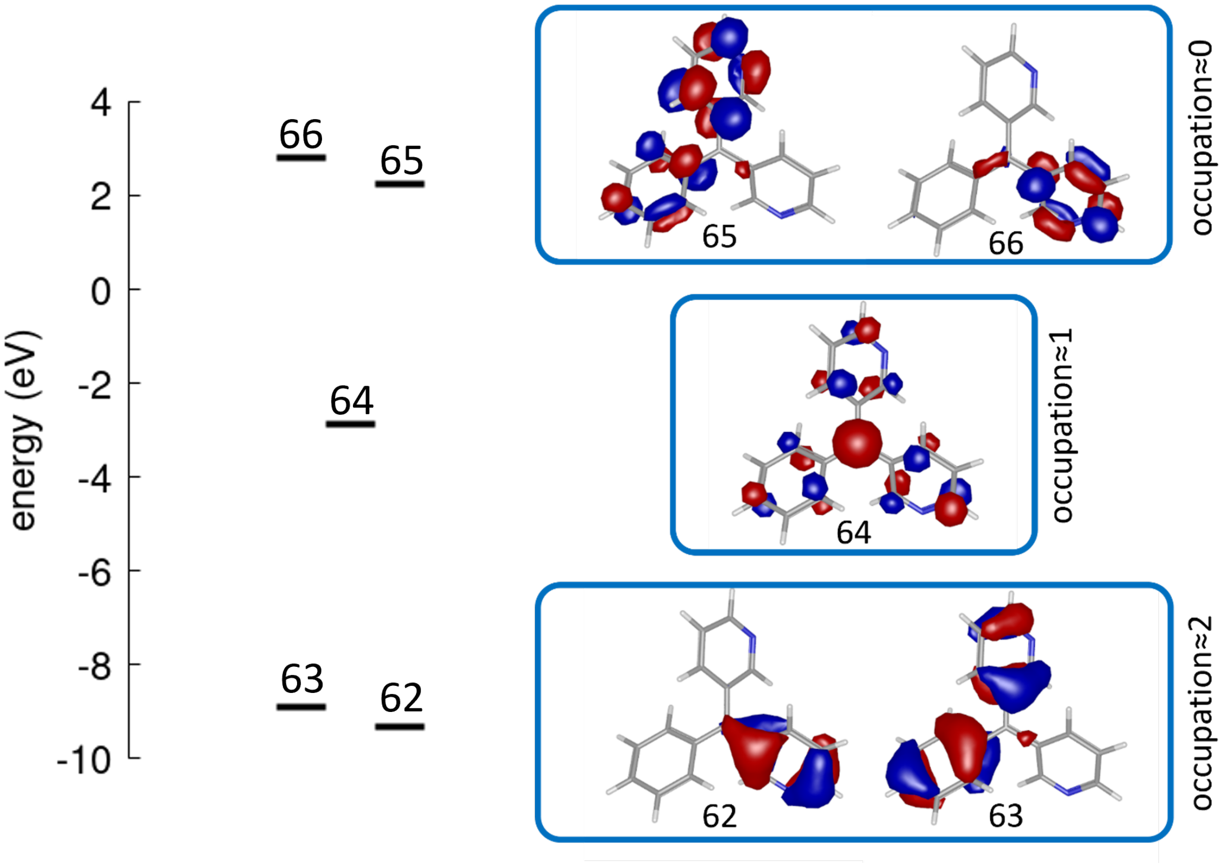

Figure S21: CASSCF frontier molecular orbitals of trityl-2N' calculated with (5,5) active space using the def2-SVP basis set.

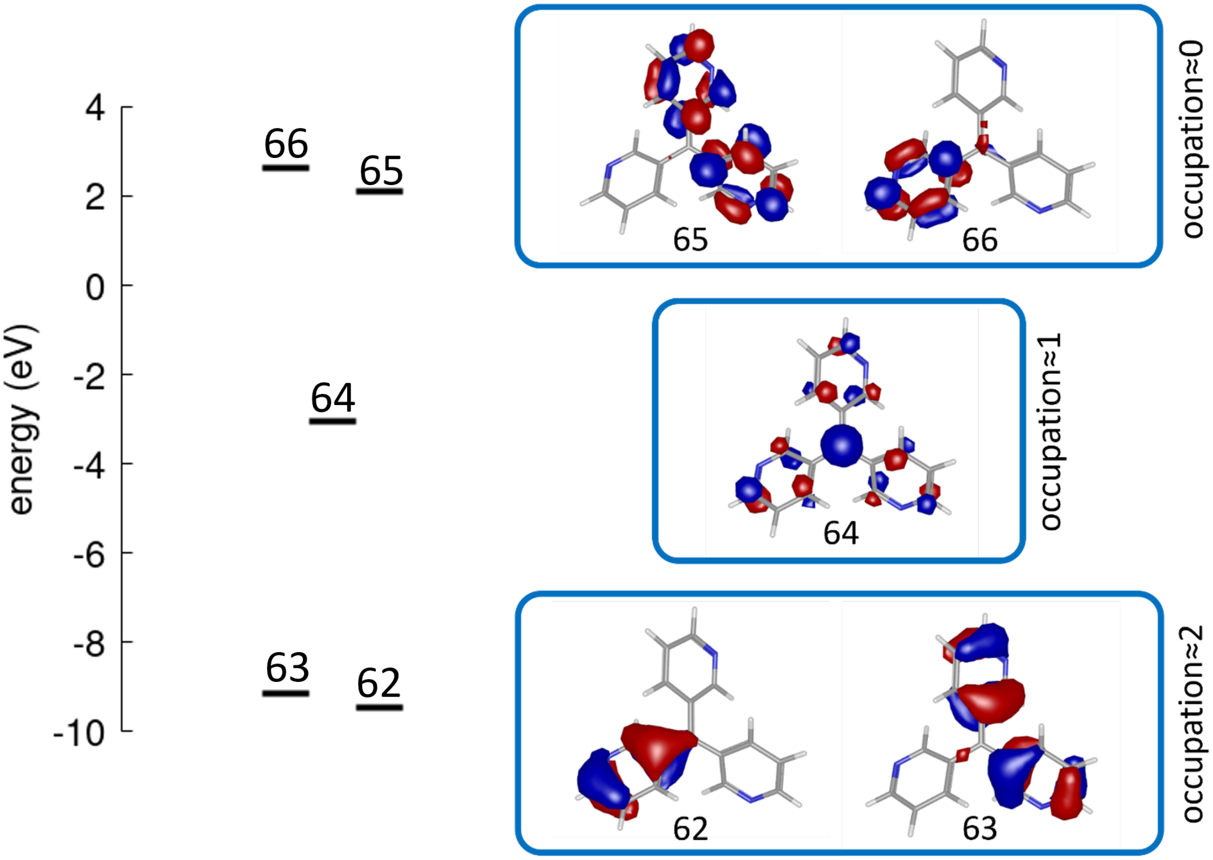

Figure S22: CASSCF frontier molecular orbitals of trityl-3N' calculated with (5,5) active space using the def2-SVP basis set.

S9 Cartesian coordinates

Table S4: Cartesian coordinates for trityl optimized geometry in the doublet ground state obtained at DFT level (UBHandHLYP/6-31G(d,p)).

atom symbol	x (Å)	y (Å)	z (Å)
C	0.1952680	-3.6987580	0.6769220
C	-0.9194690	-4.1700610	-0.0015280
C	0.4969250	-2.3503590	0.6762010
C	-0.3128300	-1.4237560	0.0001010
C	-1.7321780	-3.2723700	-0.6790610
C	-1.4376060	-1.9223120	-0.6764560
H	-2.5956770	-3.6267510	-1.2184110
C	0.0013810	0.0014020	0.0006640
C	1.3916420	0.4416440	-0.0000320
C	2.3881060	-0.2876480	-0.6691240
C	1.7880790	1.6111890	0.6690300
C	3.1065020	2.0242900	0.6702020
C	3.7043570	0.1323830	-0.6711250
H	3.3822580	2.9196120	1.2035820
H	4.4444820	-0.4418920	-1.2045660
C	4.0738730	1.2897520	-0.0007540
C	-1.0767790	0.9843390	0.0010990
C	-2.2842420	0.7425260	0.6759430
C	-0.9491170	2.2088100	-0.6743140
C	-1.9731990	3.1363920	-0.6759570
C	-3.3034340	1.6754680	0.6776500
H	-4.2140190	1.4667240	1.2156000
H	-1.8506150	4.0623140	-1.2142550
C	-3.1565510	2.8779200	0.0009750
H	-1.1518330	-5.2223690	-0.0023120
H	1.3575790	-1.9975080	1.2182450
H	-2.4073110	-0.1802410	1.2165730
H	-3.9533440	3.6034310	0.0009790
H	-0.0423140	2.4171810	-1.2158130
H	-2.0695560	-1.2391280	-1.2176250
H	5.1013350	1.6146310	-0.0010330
H	2.1142440	-1.1799290	-1.2055090
H	1.0514300	2.1835450	1.2062570
H	0.8289700	-4.3847330	1.2156720

Table S5: Cartesian coordinates for trityl-1N optimized geometry in the doublet ground state obtained at DFT level (UBHandHLYP/6-31G(d,p)).

atom symbol	x (Å)	y (Å)	z (Å)
C	-0.7593120	-4.1330050	-0.0470740
C	-0.2250920	-1.4434640	0.0090680
C	0.3589890	-3.6896900	0.6418390
C	0.6295570	-2.3371530	0.6682160
H	0.9964690	-4.3915970	1.1528210
C	0.0033060	-0.0058450	0.0015500
C	-1.1287880	0.9130430	-0.0017140
C	-2.3248040	0.6021020	0.6652250
C	-1.0642690	2.1449350	-0.6729740
C	-2.1386480	3.0133200	-0.6793860
C	-3.3948280	1.4760630	0.6613970
H	-2.0648160	3.9460570	-1.2146200
H	-4.2961730	1.2146450	1.1914820
C	-3.3104320	2.6864090	-0.0117300
C	1.3683160	0.5102730	0.0032020
C	2.4023310	-0.1585460	-0.6709920
C	1.6987280	1.6938060	0.6821490
C	2.9929790	2.1775470	0.6886530
C	3.6941270	0.3319920	-0.6677560
H	4.4646490	-0.1960950	-1.2056810
H	3.2194200	3.0822350	1.2292200
C	3.9991800	1.5017140	0.0132490
H	-1.0007380	-5.1842360	-0.0861300
H	2.1777850	-1.0581140	-1.2185180
H	5.0074470	1.8818220	0.0171200
H	0.9308470	2.2210480	1.2218450
H	1.4837860	-1.9624070	1.2060670
H	-4.1468770	3.3656820	-0.0163680
H	-2.4005460	-0.3258190	1.2059810
H	-0.1671060	2.4048460	-1.2081060
C	-1.3208300	-2.0198000	-0.6571970
H	-2.0016400	-1.3845330	-1.2012570
N	-1.5836510	-3.3136770	-0.6897440

Table S6: Cartesian coordinates for trityl-2N optimized geometry in the doublet ground state obtained at DFT level (UBHandHLYP/6-31G(d,p)).

atom symbol	x (Å)	y (Å)	z (Å)
C	-0.0012090	4.1420130	0.0003360
C	-0.0005050	1.4604030	-0.0000910
C	-0.9798050	2.2227460	0.6515490
C	0.0000450	0.0073280	-0.0000480
C	1.2665240	-0.7153830	-0.0043960
C	2.3970400	-0.2129290	0.6596020
C	1.4017220	-1.9402780	-0.6774470
C	2.6039260	-2.6206590	-0.6882740
C	3.5959140	-0.8996160	0.6512910
H	2.6823190	-3.5524410	-1.2243500
H	4.4436840	-0.4951750	1.1797690
C	3.7083880	-2.1065220	-0.0237560
C	-1.2660120	-0.7161970	0.0043250
C	-2.3966950	-0.2145870	-0.6599840
C	-1.4005790	-1.9409240	0.6777720
C	-2.6023950	-2.6220010	0.6886390
C	-3.5951820	-0.9019630	-0.6516320
H	-4.4431150	-0.4981770	-1.1803480
H	-2.6803360	-3.5536520	1.2250070
C	-3.7070470	-2.1087150	0.0237830
H	-0.0016840	5.2206070	0.0002200
H	-2.3216960	0.7107170	-1.2057660
H	-4.6430820	-2.6422850	0.0320130
H	-0.5560400	-2.3427880	1.2107240
H	-1.7728200	1.7344300	1.1959990
H	4.6447290	-2.6395540	-0.0319480
H	2.3216000	0.7124710	1.2051540
H	0.5573510	-2.3428090	-1.2101580
C	0.9781610	2.2234760	-0.6518480
H	1.7716360	1.7357330	-1.1961320
N	0.9828920	3.5443460	-0.6560540
N	-0.9852200	3.5436200	0.6561790

Table S7: Cartesian coordinates for trityl-2N' optimized geometry in the doublet ground state obtained at DFT level (UBHandHLYP/6-31G(d,p)).

atom symbol	x (Å)	y (Å)	z (Å)
C	2.8175700	3.1203100	-0.0338040
C	0.9534680	1.1102070	0.0121170
C	0.6911090	2.3174870	0.6731640
C	0.0067690	0.0039370	0.0031580
C	0.4958710	-1.3665860	0.0067630
C	1.6765040	-1.7440320	0.6604550
C	-0.1944890	-2.3948160	-0.6589510
C	2.0859660	-3.0608140	0.6283400
H	2.9844200	-3.3731790	1.1333390
C	1.3122920	-3.9831130	-0.0592930
C	-1.4269600	0.2716920	0.0018040
C	-1.9590990	1.3839440	-0.6699500
C	-2.3268220	-0.5704440	0.6741710
C	-3.6838710	-0.3112960	0.6756170
C	-3.3174280	1.6361400	-0.6710440
H	-3.6992080	2.4896680	-1.2072200
H	-4.3498180	-0.9694030	1.2094650
C	-4.1887080	0.7920410	0.0026070
H	3.5742980	3.8889940	-0.0684570
H	-1.2975200	2.0371080	-1.2131010
H	-5.2476560	0.9909520	0.0021720
H	-1.9488510	-1.4215630	1.2144370
H	-0.2345550	2.4444190	1.2083450
H	1.6029040	-5.0215540	-0.1023870
H	2.2502250	-1.0097140	1.2002090
H	-1.0981100	-2.1609380	-1.1991280
C	2.1898830	1.0282520	-0.6510540
H	2.4382770	0.1330950	-1.1993510
N	3.0922790	1.9921740	-0.6780250
N	0.1932350	-3.6564200	-0.6955550
C	1.6302080	3.3276300	0.6514650
H	1.4534140	4.2585980	1.1633490

Table S8: Cartesian coordinates for trityl-3N optimized geometry in the doublet ground state obtained at DFT level (UBHandHLYP/6-31G(d,p)).

atom symbol	x (Å)	y (Å)	z (Å)
C	-0.9477690	4.0247880	-0.0100590
C	-0.3386370	1.4141610	-0.0035210
C	0.4420020	2.3774120	-0.6578930
C	-0.0043510	0.0004190	0.0014070
C	-1.0668160	-0.9939420	0.0143470
C	-2.2885780	-0.7776170	-0.6456280
C	-0.9349300	-2.2221420	0.6763110
C	-1.9784990	-3.1234690	0.6582790
H	-1.9033590	-4.0673760	1.1713040
C	-3.1380410	-2.7883910	-0.0247390
C	1.3931610	-0.4167210	-0.0009720
C	2.3782520	0.3298750	0.6645290
C	1.8027730	-1.5806630	-0.6706720
C	3.1275370	-1.9724280	-0.6764780
C	3.7010750	-0.0688370	0.6612420
H	4.4341920	0.5175860	1.1904410
H	3.4161730	-2.8629150	-1.2106020
C	4.0848700	-1.2208500	-0.0100090
H	-1.1928040	5.0751000	-0.0124650
H	2.0938970	1.2157140	1.2067930
H	5.1173110	-1.5288070	-0.0142270
H	1.0741460	-2.1631920	-1.2082720
H	1.3252330	2.0808970	-1.2017350
H	-3.9741910	-3.4698160	-0.0565040
H	-2.4405680	0.1377840	-1.1961810
H	-0.0274260	-2.4482370	1.2098160
C	-1.4656710	1.9354790	0.6465810
H	-2.1278700	1.2829200	1.1942600
N	-1.7700910	3.2203930	0.6480000
N	0.1466920	3.6643500	-0.6653350
N	-3.2898870	-1.6379010	-0.6696630

Table S9: Cartesian coordinates for trityl-3N' optimized geometry in the doublet ground state obtained at DFT level (UBHandHLYP/6-31G(d,p)).

atom symbol	x (Å)	y (Å)	z (Å)
C	1.1180780	-4.0433070	-0.0451140
C	0.4256420	-1.3918580	0.0102840
C	-0.3113200	-2.3883290	-0.6527110
C	-0.0000880	0.0000890	0.0048720
C	0.9925730	1.0646570	0.0102150
C	2.2241640	0.9242920	-0.6524560
C	0.7836200	2.2852350	0.6659750
C	1.7658810	3.2530240	0.6388030
H	1.6296410	4.1937230	1.1449450
C	2.9429350	2.9897100	-0.0452150
C	-1.4183780	0.3273460	0.0102380
C	-2.3708250	-0.4642260	0.6658070
C	-1.9128160	1.4641760	-0.6521980
C	-3.7001420	-0.0976670	0.6387330
H	-4.4465900	-0.6862390	1.1447470
C	-4.0608260	1.0534530	-0.0450190
H	1.3593060	-5.0944150	-0.0835960
H	-2.0605030	-1.3446920	1.2025840
H	-5.0917290	1.3701290	-0.0834190
H	-1.2311650	2.0983620	-1.1969730
H	-1.2010720	-2.1149810	-1.1979030
H	3.7327480	3.7240290	-0.0836930
H	2.4323060	0.0168870	-1.1973470
H	-0.1339120	2.4568060	1.2029570
C	1.5870130	-1.8210230	0.6664000
H	2.1940610	-1.1121290	1.2036250
N	0.0162370	-3.6669950	-0.6835650
N	3.1679110	1.8471260	-0.6833140
C	1.9342520	-3.1555260	0.6392520
H	2.8169270	-3.5077780	1.1456690
N	-3.1839470	1.8198710	-0.6829480

Table S10: Cartesian coordinates for trityl-4N optimized geometry in the doublet ground state obtained at DFT level (UBHandHLYP/6-31G(d,p)).

atom symbol	x (Å)	y (Å)	z (Å)
C	2.1016340	3.5649260	0.0218940
C	0.7463880	1.2526570	0.0057930
C	0.2841760	2.4019190	0.6624550
C	0.0098970	0.0002420	0.0000080
C	0.7367170	-1.2579890	-0.0059010
C	1.9665100	-1.4334890	0.6422200
C	0.2661090	-2.4031000	-0.6636990
C	2.0737420	-3.5807760	-0.0221370
C	-1.4472190	0.0056180	0.0001240
C	-2.1712000	1.0128590	-0.6579420
C	-2.1783810	-0.9964340	0.6581500
C	-3.5596450	-0.9878630	0.6590900
C	-3.5524900	1.0142480	-0.6587940
H	-4.0827820	1.7926270	-1.1823620
H	-4.0954970	-1.7624300	1.1826530
C	-4.2554830	0.0157190	0.0001920
H	2.6469670	4.4952890	0.0284190
H	-1.6407750	1.7825680	-1.1924350
H	-5.3327970	0.0195790	0.0002200
H	-1.6534770	-1.7700340	1.1924740
H	-0.6483110	2.3786680	1.2043850
H	2.6116170	-4.5154410	-0.0285470
H	2.4124200	-0.6206450	1.1947990
H	-0.6656390	-2.3720900	-1.2065030
C	1.9782790	1.4181000	-0.6411690
H	2.4184660	0.6014290	-1.1927030
N	2.6502180	2.5547000	-0.6373240
N	0.9476240	3.5430780	0.6740880
N	2.6294700	-2.5754010	0.6384260
N	0.9206310	-3.5494260	-0.6754920

Table S11: Cartesian coordinates for trityl-5N optimized geometry in the doublet ground state obtained at DFT level (UBHandHLYP/6-31G(d,p)).

atom symbol	x (Å)	y (Å)	z (Å)
C	-2.6402050	3.1872530	-0.0088890
C	-0.9331210	1.1215850	-0.0024050
C	-0.6603280	2.3327120	-0.6531420
C	-0.0046750	0.0037970	0.0018920
C	-0.5142840	-1.3564780	0.0031240
C	-1.6990640	-1.7270060	-0.6473600
C	0.1357070	-2.4115240	0.6588480
C	-1.4571960	-3.8645070	0.0130280
C	1.4292060	0.2490060	0.0096010
C	1.9989800	1.3518560	0.6600850
C	2.3246230	-0.6109170	-0.6492820
C	3.3659190	1.5297190	0.6315680
H	3.8260660	2.3630630	1.1346590
C	4.1444060	0.6051410	-0.0490760
H	-3.3270720	4.0185310	-0.0112460
H	1.3727830	2.0437990	1.1978490
H	5.2172230	0.7129890	-0.0885990
H	1.9436130	-1.4625820	-1.1911050
H	0.2626780	2.4617980	-1.1969620
H	-1.8367840	-4.8738010	0.0174560
H	-2.2698760	-0.9960730	-1.1994630
H	1.0493270	-2.2329130	1.2044370
C	-2.1760390	1.0840660	0.6438150
H	-2.4801960	0.2047940	1.1908220
N	-3.0205470	2.0985970	0.6442990
N	-1.4972120	3.3533800	-0.6595650
N	-2.1679410	-2.9608250	-0.6463800
N	-0.3250100	-3.6480790	0.6678190
N	3.6333750	-0.4441900	-0.6817000

Table S12: Cartesian coordinates for trityl-6N optimized geometry in the doublet ground state obtained at DFT level (UBHandHLYP/6-31G(d,p)).

atom symbol	x (Å)	y (Å)	z (Å)
C	-0.0612200	4.1310080	-0.0000210
C	-0.0215430	1.4523160	0.0000440
C	0.9500780	2.2276830	-0.6476330
C	0.0000040	-0.0001050	0.0000850
C	-1.2470630	-0.7449030	0.0000440
C	-2.4044620	-0.2907590	-0.6472190
C	-1.3962030	-1.9792830	0.6471740
C	-3.5471300	-2.1184100	-0.0000720
C	1.2686040	-0.7075970	0.0000510
C	2.4121290	-0.2193450	0.6471560
C	1.4541750	-1.9370420	-0.6470430
C	3.6083580	-2.0123890	-0.0000310
H	-0.0771760	5.2091230	-0.0000190
H	2.3753570	0.7113430	1.1924350
H	4.5499920	-2.5376720	0.0001600
H	0.6431160	-2.3948310	-1.1924510
H	1.7517940	1.7540280	-1.1933230
H	-4.4727880	-2.6713610	-0.0003650
H	-2.3951870	0.6405950	-1.1925220
H	-0.5719810	-2.4128580	1.1926420
C	-1.0157230	2.1985910	0.6476590
H	-1.8031030	1.7014230	1.1933090
N	-1.0396780	3.5178520	0.6510370
N	0.9349590	3.5470720	-0.6510680
N	-3.5395120	-0.9634760	-0.6506820
N	-2.5267620	-2.6595410	0.6504530
N	2.6043900	-2.5835130	-0.6504390
N	3.5665950	-0.8581640	0.6505510

References

(1) Nakano, M.; Champagne, B. Theoretical Design of Open-Shell Singlet Molecular Systems for Nonlinear Optics. *The Journal of Physical Chemistry Letters* **2015**, *6*, 3236–3256.

(2) Nakano, M.; Champagne, B. Nonlinear optical properties in open-shell molecular systems. *WIREs Computational Molecular Science* **2016**, *6*, 198–210.

(3) Frisch, M. J. et al. Gaussian~16 Revision B.01. 2016; Gaussian Inc. Wallingford CT.

(4) Franz, M.; Neese, F.; Richert, S. Calculation of exchange couplings in the electronically excited state of molecular three-spin systems. *Chemical Science* **2022**, *13*, 12358–12366.

(5) Neese, F. The ORCA program system. *WIRES Comput. Molec. Sci.* **2012**, *2*, 73–78.

(6) Surján, P. R. *Second Quantized Approach to Quantum Chemistry*; Springer Berlin Heidelberg, 1989.

(7) Ramasesha, S.; Soos, Z. Magnetic and optical properties of exact PPP states of naphthalene. *Chemical Physics* **1984**, *91*, 35–42.
